# MORF4L2 induces immunosuppressive microenvironment and immunotherapy resistance through GRHL2/MORF4L2/H4K12Ac/CSF1 axis in triple-negative breast cancer

**DOI:** 10.1186/s40364-024-00719-1

**Published:** 2025-01-09

**Authors:** Xin-Yi Sui, Shuo-Wen Cao, Xiao-Qing Song, Xi-Yu Liu, Chao Chen, Qingya Yan, Zhi-Qing Wang, Wen-Juan Zhang, Lin-Xiaoxi Ma, Xi Jin, Ding Ma, Yi Xiao, Song-Yang Wu, Ying Xu, Zhi-Ming Shao, Lei Fan

**Affiliations:** 1 Department of Breast Surgery, Fudan University Shanghai Cancer Center, Shanghai, China; 2 Key Laboratory of Breast Cancer in Shanghai, Department of Oncology, Shanghai Medical College, Fudan University, Shanghai, China; 3 School of Basic Medical Sciences, Xinxiang Medical University, Xinxiang, China

**Keywords:** Triple-negative breast cancer, Mortality factor 4 like 2, Anti-PD1, Tumor microenvironment, Histone 4 lysine 12 acetylation, Combination therapy

## Abstract

**Background:**

Although immunotherapy has achieved great progress in advanced triple-negative breast cancer (TNBC), there are still numerous patients who do not benefit from immunotherapy. Therefore, identification of the key molecule that induces immune escape and clarification of its specific mechanism in TNBC are urgently needed.

**Methods:**

In this research, single cell sequencing and bulk sequencing were conducted for biomarker screening. Immunohistochemistry, multiplex immunofluorescence, and orthotopic TNBC tumor model were applied in identifying the key molecule driving immune escape. At the mechanical level, RNA sequencing, in vitro co-culturing system, flow cytometry, Western blotting, ELISA, and real-time qPCR were carried out.

**Results:**

Mortality factor 4 like 2 (MORF4L2) expression is significantly up-regulated among patients who developed anti-PD1 resistance. MORF4L2 enhances anti-PD1 resistance by inducing the chemotaxis of macrophage infiltration and promoting their polarization towards the alternative activation phenotype (M2), thus creating an immunosuppressive microenvironment. Mechanistically, MORF4L2 actes as part of NuA4 histone acetyltransferase (HAT) complex, contributes to to histone 4 lysine 12 acetylation (H4K12Ac) and activates the downstream transcription of macrophage colony-stimulating factor (CSF1). CSF1 is secreted by tumor cells and binds to the macrophage-surface CSF1 receptor (CSF1R), which chemotactically converted and polarized macrophages to the M2 phenotype. Furthermore, we revealed that grainyhead like transcription factor 2 (GRHL2) could promote MORF4L2 transcription by binding to the MORF4L2 enhancer region. Notably, BLZ549, an inhibitor of CSF1R, restored the anti-PD1 sensitivity by blocking the GRHL2/MORF4L2/H4K12Ac/CSF1 axis.

**Conclusions:**

GRHL2/MORF4L2/H4K12Ac/CSF1 axis plays an important role in anti-PD1 resistance. CSF1R inhibitors can reverse GRHL2/MORF4L2-mediated anti-PD1 resistance.

**Supplementary Information:**

The online version contains supplementary material available at 10.1186/s40364-024-00719-1.

## Background

Breast cancer shows the highest morbidity among female malignancies globally [1, 2]. Particularly, triple-negative breast cancer (TNBC) is associated with worst prognosis among all molecular subtypes due to the lack of effective therapeutic targets [3, 4]. Recently, immunotherapy has emerged as a novel promising therapeutic option [5, 6]. As demonstrated in the KEYNOTE-355, IMpassion131, and TORCHLIGHT trials, immunotherapy significantly improves the prognosis of patients with advanced first-line TNBC [7–9]. Additionally, its unique long tailing effect can help patients achieve good outcomes consistently over a long period of time [10]. However, anti-programmed cell death protein-1 (PD1) therapy has achieved limited efficacy in patients with negative results in programmed cell death-ligand 1 (PD-L1) immunohistochemical analysis. Furthermore, many patients still develop primary or secondary resistance regardless of the PD-L1 status [11, 12]. Consequently, exploring the immunotherapy resistance mechanisms and optimizing immunotherapy regimens are highly necessary.

Stephen Paget proposed the theory of "seed and soil" in 1889, in which cancer cells are regarded as seeds that only survive in a suitable microenvironmental soil [13]. Macrophages, as the largest component of the microenvironment, play a critical role in tumorigenesis and development [14]. Notably, macrophages exert a dual effects on tumors [15]. In general, macrophages of M1 phenotype exhibit the pro-inflammatory capacity and can suppress cancer cells, whereas those of M2 phenotype possess similar functions to tumor-associated macrophages (TAMs) and contribute to tumor progression via a variety of pathways, including promotion of tumor growth, migration, and suppression of immune responses [16]. In addition, studies have reported that macrophages can also drive immunotherapy resistance [17]. Taken together, although macrophages have been reported to affect the anti-tumor immunity, the underlying mechanisms are unclear, and it is still needed to identify the upstream drivers that regulate TAMs [18–20].

In the current study, we utilized pre-treatment transcriptomic analysis of tumor tissue from patients receiving immunotherapy to identify genes indicative of immunotherapy resistance. In our cohort, high Mortality factor 4 like 2 (MORF4L2) expression was identified as suggestive of poor immunotherapy efficacy. Previous studies have revealed that MORF4L2 may be associated with the immunosuppressive microenvironment [21]. MORF4L2 proteins are involved in chromatin modification, gene regulation, and DNA repair [22, 23]. Our study revealed that MORF4L2 promotes colony stimulating factor 1 (CSF1) expression, which in turn stimulates macrophage polarization, leading to anti-PD1 resistance. Grainyhead like transcription factor 2 (GRHL2), a DNA-binding protein, has been reported to be involved in the transcriptional regulation of several genes [24]. At the molecular level, MORF4L2 expression is regulated by GRHL2 and participates in the formation of the NuA4 histone acetyltransferase (HAT) complex, which promotes the transcriptional activation of CSF1. Consequently, blocking CSF1 inhibits TNBC anti-PD-1 treatment resistance. These findings reveal the mechanism by which GRHL2/MORF4L2 promotes TNBC anti-PD-1 treatment resistance and suggest potential therapeutic targets.

## Methods

### Datasets

The advanced TNBC clinical trial dataset (SPARK, NCT04734262) was utilized in this study. In total, RNA sequencing data were collected from 23 patients and examined. Six individuals (including 5 metastatic samples from patients receiving immunotherapy and 1 paired primary sample) also underwent single-cell sequencing. Methods for biological sample collection and RNA sequencing are described in detail in our previous studies [25, 26]. Clinical response was evaluated using the Response Evaluation Criteria in Solid Tumors (RECIST, v1.1). In addition, The Cancer Genome Atlas (TCGA, https://tcga-data.nci.nih.gov/tcga/) and Fudan University Shanghai Cancer Center (FUSCC) databases were utilized. All clinical samples were collected under the approval of the FUSCC Ethics Committee, and the patients provided informed consents. This work was performed following the Declaration of Helsinki [27].

### RNA sequencing

Fresh frozen tissues were collected from 23 patients prior to treatment with anti-PD1 antibodies. In addition, cells were also harvested from the MORF4L2 knockdown group and the control group (SUM159) for RNA sequencing. RNA was then extracted with RNeasy mini kit (Qiagen, Germany) and sequenced on Illumina HiSeq platform. Reads were mapped to the Homo sapiens genome (hg19) for comparison. DEGs were identified using the R package "limma". The hallmark gene sets downloaded from MSigDB were used for pathway enrichment analysis with the gene set enrichment analysis (GSEA) software. Additionally, single sample gene set enrichment analysis (ssGSEA) was conducted for calculating immune cell infiltration score by calculating the ssGSEA score corresponding to each gene set.

### Cell lines

Briefly, MDA-MB-231, SUM159, MDA231-LM2-4175, HCC1806, MDA-MB-468, 4T1, AT3 and HEK293T cells were provided by the American Type Culture Collection, cultivated within Dulbecco’s modified eagle medium (DMEM) that contained fetal bovine serum and penicillin–streptomycin, and incubated at 37 °C with 5% CO_2_. To maintain the quality and reliability of these cell lines, cells within 6 passages after acquisition were used. In addition, the cells were regularly tested for mycoplasma contamination.

### Plasmid construction and transfection

Cell transfection was performed using small interfering RNA (siRNA) from Ribobio (Guangzhou, China). The lipofectamine RNAiMAX transfection reagent (Thermo Fisher Scientific) was applied to transfect siRNA, and knockdown efficiency was assessed using reverse transcription polymerase chain reaction (RT-qPCR) and Western blotting at 48 h post-transfection.

The short hairpin RNA (shRNA) plasmid was provided by Genechem (Shanghai, China). The target sequences are shown in the Supplementary Information (Table S1). To generate MORF4L2 overexpression plasmid, PCR-amplified full-length cDNA was inserted into the pCDH vector using the following primers 5-GATTCTAGAGCTAGCGAATTCATGAGTTCCAGAAAGCAGGGTTC-3 and 5-ATCCTTCGCGGCCGCGGATCCTCACAGGGCTTTGCGGTGG-3. Later, Lipofectamine 2000 (Thermo Fisher Scientific, Waltham, MA, USA) was used to transfect target plasmids and packaging plasmids (psPAX2 and pMD2.G) in HEK293T cells, then the lentivirus-containing cell supernatants were collected and passed through the 0.45 μm filters. Subsequently, lentivirus was infected in target cells, and cells were further cultured for 1 week with 1 μg/mL puromycin to obtain the stably knocked-down target cells.

### RT-qPCR assay

The Fast All-in-One RT Kit (ESScience, Shanghai, China) was adopted for extracting total cellular RNA, which was later prepared to cDNA with HiScript IV RT SuperMix for qPCR kit (Vazyme, Nanjing, China). AceQ Universal SYBR qPCR Master Mix (Vazyme, Nanjing, China) was utilized for RT-qPCR. Supplementary Information (Table S2) displays primer sequences.

### Immunoblotting assay

The cells were lysed with the RIPA cell lysis buffer (Beyotime, Shanghai, China). Thereafter, the BCA Protein Assay Kit (Solarbio, Beijing) was adopted for determining protein content. After centrifugation, the supernatant was aspirated, and added into the 5X loading buffer (Biosharp, Anhui), followed by 10-min boiling under 99˚C. Subsequently, proteins (10–20 μL) were injected into the 10%-15% sodium dodecyl sulfate—polyacrylamide gel electrophoresis (SDS-PAGE) reaction wells for separation, followed by transfer onto the 0.22-μm polyvinylidene difluoride (PVDF) membrane. After blocking using 5% skimmed milk, the membrane was washed with tris buffered saline with tween (TBST) and probed using primary antibody overnight under 4 °C. The membrane was later rinsed thrice with TBST (10 min each), prior to 60-min incubation using species-matched secondary antibody (Jackson, USA) under ambient temperature. Visualization was performed with the electrogenerated chemiluminescence (ECL) imaging system (Tanon, Shanghai, China). Antibodies are listed in the Supplementary Information (Table S3).

### Clonogenic cell survival assay

Approximately 200 4T1 cells were counted using a cell counter and placed into a 6-well plate. After 2 weeks, 0.25% crystal violet methanol solution (Solarbio, Beijing, China) was added for cell staining and then colonies including ≥ 50 cells were counted. Three separate assays were carried out for ensuring the result reliability.

### Cell counting kit-8 (CCK-8) assay

A cell suspension containing 1,000 cells/100 μL DMEM was spread in a 96-well plate. Later, 10 μL CCK8 solution (Selleck, Shanghai, China) was introduced into every well at a fixed time point each day for 4 consecutive days. The optical density was detected at 450 nm following 2-h incubation under 37 °C. Three biological replicates were set to ensure the reliability of the data.

### Eznyme linked immunosorbent assay (ELISA)

The supernatant of cells after 24-h culture within serum-free DMEM was collected, then cells suspended in the supernatant were removed using a 0.45-mm filter. The presence of CSF1 cytokines in the cell supernatant was detected using a human ELISA kit (Aiyou Biotechnology Center, Shanghai, China) in line with specific protocols.

### Co-culture assays

Co-culture assays were designed to verify polarization and recruitment of macrophages. To be specific, the macrophage polarization assay was carried out with Transwell chambers (pore size, 0.4 μm; Corning, NY, USA). Briefly, THP-1 cells were induced with PMA for 24 h to obtain M0 macrophages. Then, 5 × 10^5^ treated THP-1 cells or RAW246.7 cells were added to the lower chamber, whereas 5 × 10^5^ SUM159 cells or 4T1 cells were inoculated in upper chamber and cultured for 48 h. Finally, THP-1 cells or RAW246.7 cells were collected from the lower chamber for RT-qPCR analysis.

In the macrophage migration assay, 5 × 10^5^ SUM159 cells or 4T1 cells were first added into the lower chamber of a 24-well plate supplemented with 1000 μL serum-containing DMEM. Afterwards, THP-1 cells treated with PMA for 24 h were placed in the upper chambers (pore size, 8 μm; Corning, NY, USA), and then 600 μL serum-free DMEM was added. After 48-h incubation under 37 °C, cells were stained using 0.25% crystal violet methanol solution (Solarbio, Beijing, China). Finally, cells were randomly photographed under an inverted microscope by selecting three fields of view.

### Multiple immunofluorescence (mIF) analysis

The mIF staining was carried out with the Four-color Fluorescence Kit (Recordbio Biotechnology, Shanghai, China) according to manufacturer’s instruction. The antibodies used are listed in the Supplementary Information. Images were detected and captured using the CaseViewer software.

### Immunohistochemistry (IHC) assay

Breast cancer tissue samples were harvested from patients for IHC staining using the MORF4L2 primary antibody. In brief, samples were subjected to fixation within 10% formalin and paraffin embedding. Later, 4-μm sections were prepared with a microtome prior to mounting onto the glass slides, followed by xylene deparaffinage and gradient alcohol rehydration. Sections were then heated within citrate buffer (pH 6.0) in the autoclave to achieve antigen retrieval. Hydrogen peroxide was added to block endogenous peroxidase activity. Afterwards, the primary antibody against MORF4L2 (1:100) was added for overnight section incubation under 4 °C. Afterwards, sections were washed and probed using a horseradish peroxidase (HRP)-labeled secondary antibody for 30 min under ambient temperature. The antigen–antibody complexes were observed using the 3,3'-diaminobenzidine (DAB) substrate. Results were integrated by two independent pathologists and scored for MORF4L2 expression.

### Animal studies

The 5–6-week-old BALB/c female mice and C57BL/6 female mice were provided by GemPharmatech (Jiangsu, China). The mice were treated strictly following the Principles of Vertebrate Utilization and the Care and the Guidelines for the Management of Laboratory Animal Feeding in China. Our experimental protocols gained approval from the FUSCC Animal Care and Use Committee.

Firstly, we injected 1 × 10^6^ 4T1 or AT3 tumor cells in mice to construct the orthotopic tumor xenograft models. Secondly, the tumor size was determined using a vernier caliper 2–3 times per week. When the tumor volume grew to 2,000 mm^3^ or the mice lost more than 20% of their own body weight, the experiments were terminated.

For drug treatment, animals were randomized as four diverse treatment groups after the tumors were palpable. Each mouse was then intraperitoneally injected with 200 μg anti-PD1 antibody every three days or with anti-IgG at three-day intervals (Bio X Cell, West Lebanon, NH, USA). Mice were also given oral administration of BLZ945 at 100 mg/kg (Selleck, Houston TX, USA) daily. The vernier caliper was utilized for measuring tumor size 2–3 times weekly, and the experiments were stopped after tumor volume exceeded 2000 mm^3^ or the mice lost more than 20% of their own body weight. Tumor volume was determined below:

$$\text{tumor volume }=0.5 \times \text{L }\left(\text{length}\right)\times \text{W }{(\text{width})}^{2}$$

### Flow cytometry

Tumor tissues were collected from mice, and cells were digested with enzymes (0.5 mg/mL type I collagenase (Sigma-Aldrich), 1 mg/mL Dispase (Roche) and 1 mg/mL hyaluronidase (Sigma-Aldrich)) under 37 °C for a 1-h duration into single cell suspensions. These cells were then passed through the 100-µm and 40-µm filters, respectively. After lysis of the erythrocytes, cells were incubated with flow-through antibodies (Supplementary Material) for 30 min in dark at 4 °C. Eventually, CytExpert software (Beckman Coulter) was used for data analysis.

### Chromatin immunoprecipitation (ChIP)

ChIP was performed on tumor cells. Samples were prepared using the SimpleChIP Plus Enzymatic Chromatin IP Kit according to the manufacturer's instructions (Cell Signaling Technology #9005, Shanghai, China). Antibody and primers used for ChIP-qPCR are in the Supplementary Material (Table S3 and Table S4).

### Genomic sequencing data analysis

Data of H4K12Ac were obtained from the Gene Expression Omnibus (GEO, https://www.ncbi.nlm.nih.gov/geo/) database (GSE107749 and GSE65886). Data for grainyhead like transcription factor 2 (GRHL2) ChIP-sequencing were obtained from the GEO database (GSE109820 and GSE81714). The raw data was submitted to the trim-galore tool for reads filtration. Later, the cleaned reads were further subjected to alignment with bowtie2 using hg19 assembly and deduplicated by picard tools. For data visualization, the deduplicated reads were subjected to the build index file with samtools and converted into the bigwig file with settings “–normalizeUsing RPGC –effectiveGenomeSize 2864785220 –ignoreDuplicates”.

### Coimmunoprecipitation (Co-IP)

Cells were initially collected and washed twice with pre-cooled PBS. Pre-cooled NP-40 lysis buffer lysis was used to lyse the cells while PMSF was added [28]. The supernatant is collected by centrifugation at 12,000g for 5 min at 4 °C and incubated overnight with primary antibody (anti-MORF4L2) or IgG in a rotary incubator at 4 °C (Table S3). Samples were then incubated with protein A/G beads (Bimake, Shanghai, China) for an additional 2 h. Immunoprecipitates were washed 3 times and then boiled for 5 min to denature them for subsequent immunoblot analysis.

### Dual luciferase assay

After amplification of the MORF4L2 enhancer, the PCR product was cloned into the pGL3-Basic vector. DNA sequencing was used to confirm the plasmid sequence. HEK293T and SUM159 cells were inoculated in 24-well plates and cultured to a cell density of 60%. Cells were transfected in triplicate with the promoter reporter gene plasmid, the pRL-β-actin internal control plasmid, and siGRHL2 or negative. Forty-eight hours after transfection, cells were collected for fluorescence assay (Dual Luciferase Reporter Assay Kit, Vazyme, Nanjing, China).

### Single cell RNA sequencing (scRNA-seq)

Patient biopsies were collected, then, the single-cell libraries were created using Chromium Single Cell 3' Reagent Kits (10 × Genomics) and sequenced using the Illumina sequencing platform (NovaSeq). “Cell Ranger” was employed to process the raw data and map them to the GRCh38 human genome. Cells were later screened using the R package "Seurat" by removing those expressing < 300 or > 7000 genes and those with > 10% mitochondrial count. The dataset was corrected for bulk effects by an anchor-based canonical correlation analysis (CCA) method. Subsequently, data were downscaled using the uniform manifold approximation and projection (UMAP) method [29] and cells were clustered based on previous studies [30]. Moreover, R software packages "clusterProfiler", "GSVA", and Molecular Signatures Database (MSigDB) were utilized for gene set variation analysis (GSVA) and pathway enrichment analysis. To explore the cell–cell interaction niches in tumor tissues, cell communication analysis was performed with "CellChat" [31]. Also, the "monocle2" package was adopted for pseudotime analysis. Developmental initiation sites were identified based on biological significance.

### Statistical analysis

Kaplan–Meier and log-rank tests were adopted for plotting the survival curves and making statistical comparisons. Additionally, Cox proportional risk regression was applied in univariate analysis. Two-tailed Student's t-tests, analysis of variance (ANOVA), Spearman's correlation test, Wilcoxon's test, and Pearson's χ^2^ test were performed using R or GraphPad Prism software. *P* < 0.05 stood for statistical significance (ns = not significant, *P < 0.05, **P < 0.01, ***P < 0.001).

## Results

### The DNA repair pathway is associated with unfavorable prognosis among TNBC patients receiving immunotherapy

**Fig. 1:**
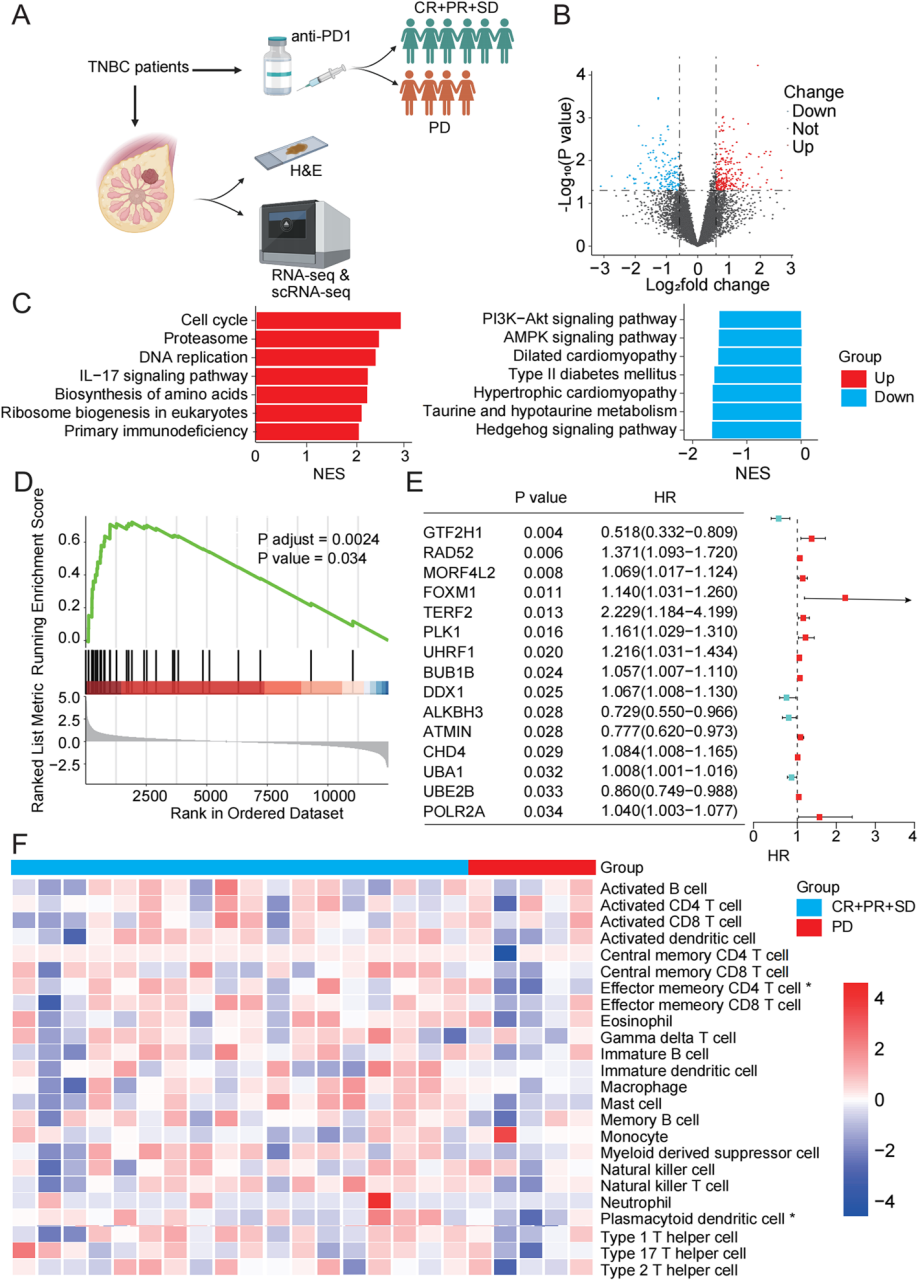
DNA repair pathway and MORF4L2 are strongly associated with anti-PD1 treatment resistance in TNBC. **A** Schematic diagram of patient recruitment and specimen collection. **B** Volcano plot of DEGs among patients with different efficacy (PD vs CR + PR + SD). **C**-**D** Pathway enrichment analysis of DEGs. **E** Forest plot demonstrating the results of univariate analysis of genes associated with DNA repair pathways. **F** Analysis of immune cell infiltration among patients with different efficacy. MORF4L2 mortality factor 4 like 2; TNBC triple-negative breast cancer; DEGs differentially expressed genes; PD progressive disease; CR complete response; PR Partial response; SD stable disease

Tumor tissues were collected from advanced TNBC patients treated with immunotherapy for next-generation sequencing (SPARK, NCT04734262). A total of 62 patients were included in the SPARK trial, and bulk sequencing data were collected from 23 of them. The basic features of these 23 cases were consistent with those of all patients (Table 1). Patients were classified into disease progression (PD, *n* = 5) and others (complete response (CR) + partial response (PR) + stable disease (SD), *n* = 18) groups according to the disease control rate (DCR) (Fig. 1A). Additionally, differentially expressed gene (DEG) analysis was conducted to define differences in mRNA expression between patients with different efficacy (Fig. 1B). Notably, based on gene set enrichment analysis (GSEA), DNA repair pathway was significantly related to patients with poor efficacy (Fig. 1C, D). Subsequently, through literature review, the set of genes associated with DNA repair was identified (Table S5) [27], among which, MORF4L2 was discovered as a key molecule affecting prognosis by univariate Cox regression analysis (Fig. 1E). After integrating other characteristics, multivariate Cox regression also revealed that MORF4L2 was related to a worse outcome (Table 2). Additionally, the immune microenvironmental cells were explored among patients with different efficacies. As a result, patients with efficacy assessed as PD presented a relatively cold immune microenvironment (Fig. 1F).

**Table 1:**
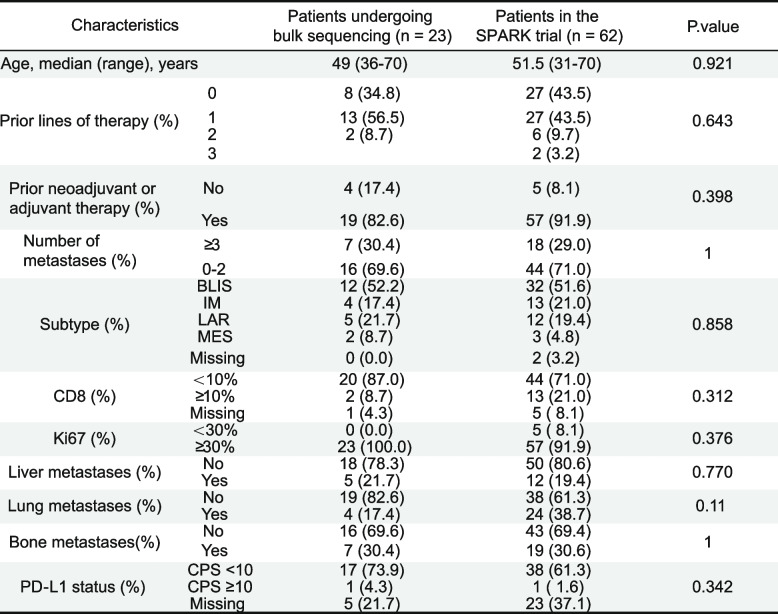
Demographic and baseline clinical characteristics

**Table 2:**
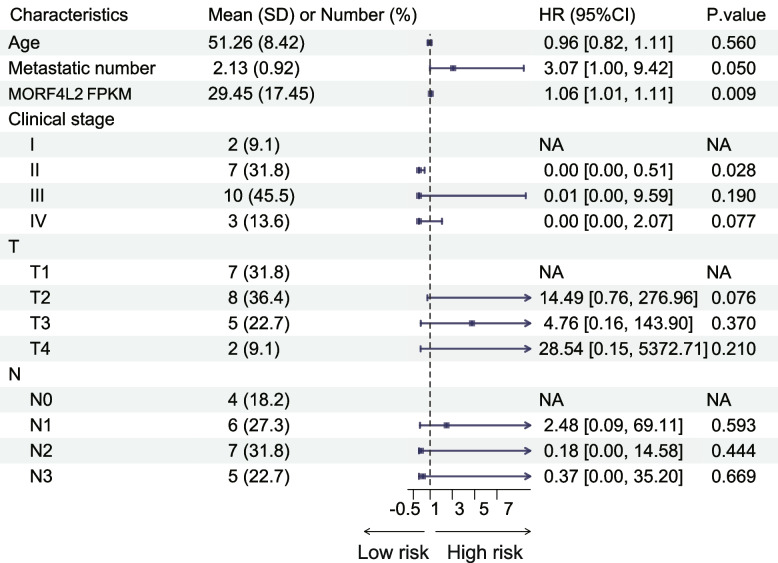
Multivariate analysis for PFS outcomes

*Abbreviations*: *PFS *progression-free survival, *SD *standard deviation, *HR *hazard ratio, *CI *confidence interval, *FPKM *fragments per kilobase million, *T *tumor size, *N *lymph node

### ScRNA-seq reveals that the DNA repair pathway is essential for the resistance to anti-PD1 therapy

**Fig. 2:**
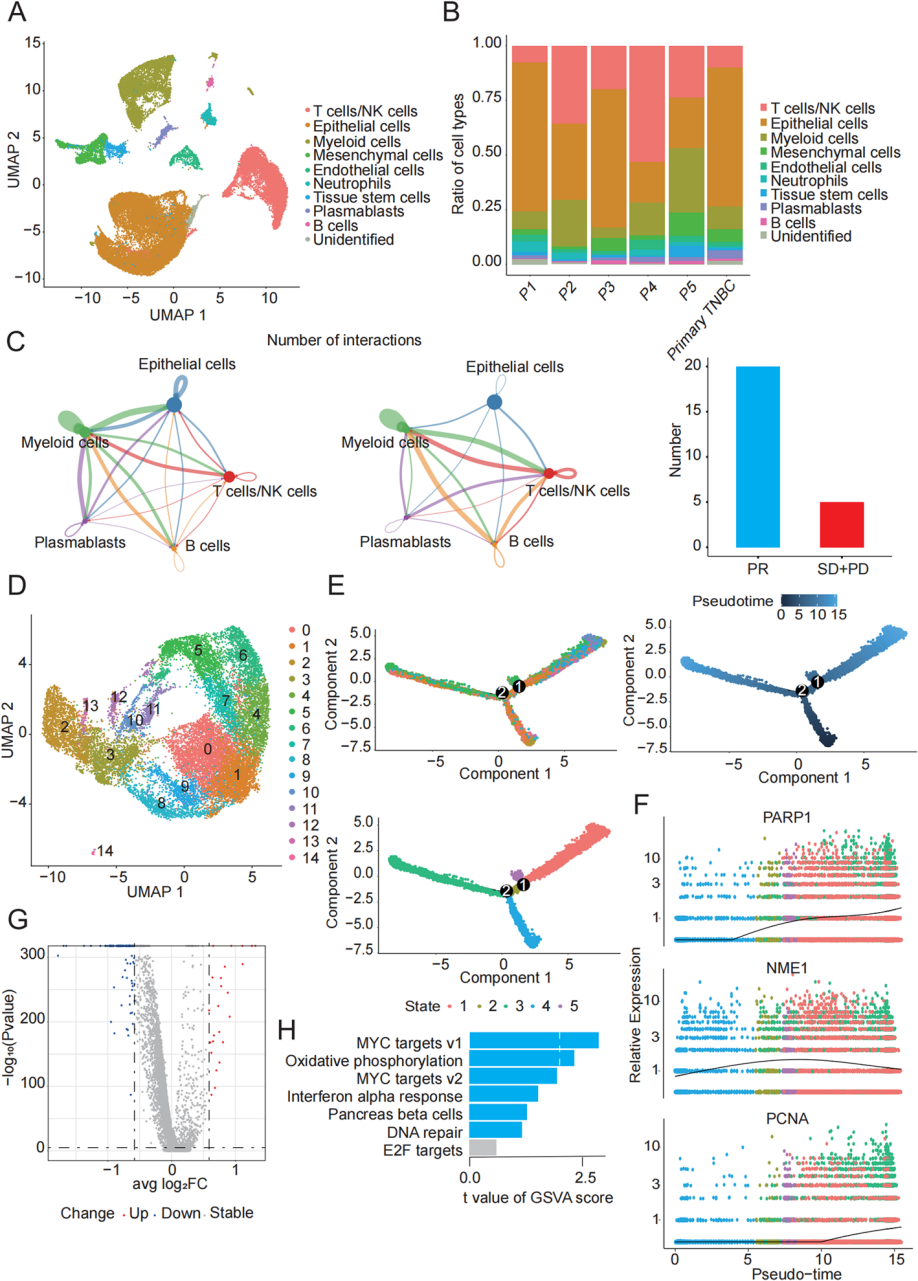
Characterization of efficacy-associated microenvironmental cells. **A**, **B** UMAP plots of tumor tissue from the six patients in this study, colored by major cell type. **C** Analysis of interactions between lymphocytes and tumor cells. Arrows and edge colors indicate direction. Circle size is proportional to the number of cells in each cell group. Edge thickness indicates the number of interactions between populations. Bar graph indicates the number of cell-to-cell interactions. **D** UMAP plot of epithelial cells with each cluster displayed in a different color. **E** Potential trajectory analysis of epithelial cells. **F** Dot plot of dynamic expression of key genes for DNA repair. **G**, **H** Volcano plot and GSVA pathway enrichment analysis of epithelial DEGs among patients with different efficacy (PD vs CR + PR + SD). *P < 0.05, **P < 0.01, ***P < 0.001. UMAP uniform manifold approximation and projection; DEGs differentially expressed genes; GSVA genomic variation analysis; PD progressive disease; CR complete response; PR Partial response; SD stable disease

To comprehensively analyze the cellular composition of the microenvironment among patients with different outcomes, scRNA-seq was performed on metastatic samples collected by pre-treatment punctures from five patients and one paired sample from an advanced primary tumor. After filtering out potentially damaged and dead cells, UMAP clustering was carried out on these cells and nine cell types were detected, including T/NK cells, epithelial cells, myeloid cells, mesenchymal cells, endothelial cells, neutrophils, tissue stem cells, plasmablasts, and B cells (Fig. 2A, B and Figure S1A-B). The potential interactions of tumor cells with immune cells were analyzed initially. CellChat analysis revealed a lower amount of interactions of tumor cells with immune cells among patients achieving PD (Fig. 2C). As the tumor cells were of epithelial origin, we conducted high-resolution UMAP analysis on epithelial cells and 14 clusters were redefined (Fig. 2D). Furthermore, the trajectory analysis was performed according to Monocle2, which defined the primary tumor as a developmental initiation point based on biological meaning (Fig. 2E). The DNA repair pathway plays a role in tumor dissemination, and molecules such as poly (ADP-ribose) polymerase 1 (PARP1), NME/NM23 nucleoside diphosphate kinase 1 (NME1) and proliferating cell nuclear antigen (PCNA) are highly expressed in metastatic lesions (Fig. 2E, F). In addition, DEGs were identified in tumor cells from patients with different efficacy (PD, n = 3; CR + PR + SD, n = 2, Figure S1C, Table S6), and GSVA of DEGs was carried out based on MsigDB to search for pathways that might be related to immunotherapy efficacy (Fig. 2G, H). The most significantly enriched pathways included MYC targets v1, MYC targets v2, oxidative phosphorylation, interferon alpha response, pancreas beta cells, and DNA repair. Taken together, our finding revealed that the DNA repair pathway might be positively related to anti-PD1 resistance and tumor progression of TNBC.

### MORF4L2 is a key molecule that induces the immunosuppressive microenvironment

**Fig. 3:**
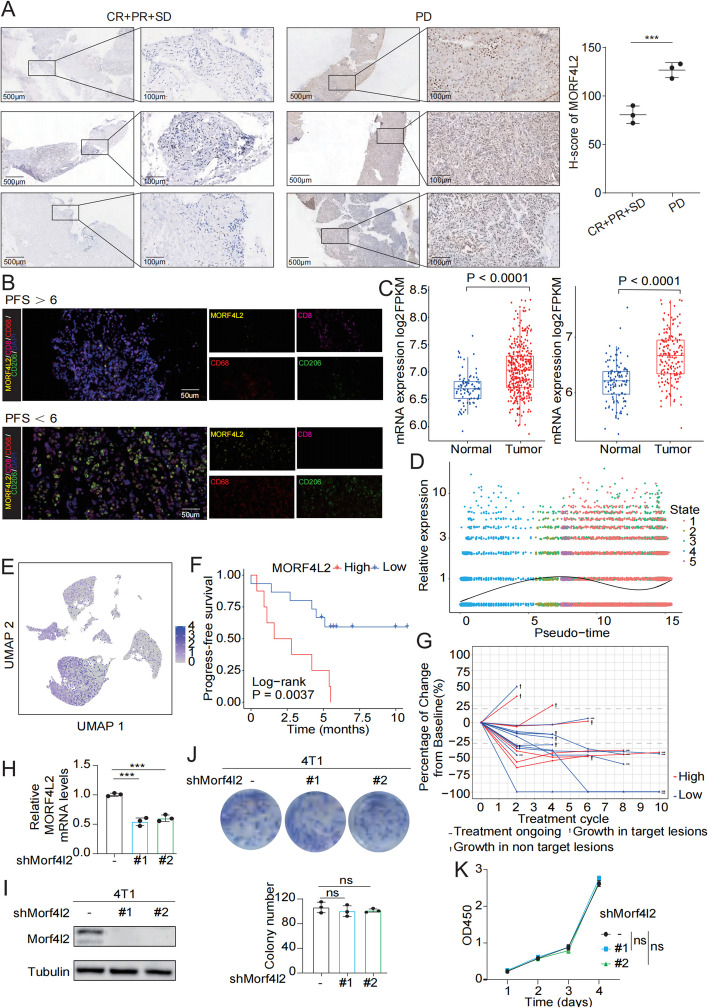
MORF4L2 induces suppression of the immune microenvironment. **A** IHC staining was performed with MORF4L2-specific antibodies to verify the predictive value of MORF4L2 protein expression for anti-PD1 efficacy. **B** Representative images of MORF4L2 protein detected together with other immune cells by using mIF. Yellow (MORF4L2 cells), pink (CD8 cells), red (CD68 cells), green (CD206 cells). **C** Expression of MORF4L2 mRNA in normal and tumor tissues was analyzed utilizing FUSCC (left) and TCGA (right) databases. **D** Dot plot of MORF4L2 mRNA dynamic expression. **E** UMAP analysis of MORF4L2 expression in tumor and microenvironmental cells. **F** PFS curve based on MORF4L2 expression was analyzed by log-rank test using the Kaplan–Meier method. **G** Spider graph depicted the relationship between MORF4L2 expression and percentage change in tumor size at different time points. **H**, **I** RT-qPCR and immunoblotting were used to analyze the knockdown efficiency of MORF4L2. **J**, **K** Clonogenic cell survival assays and CCK8 were performed with 4T1 cells. MORF4L2 mortality factor 4 like 2; mIF multiple immunofluorescence; FUSCC Fudan University Shanghai Cancer Center; TCGA the Cancer Genome Atlas; UMAP uniform manifold approximation and projection; PFS progression-free survival; CCK8 cell counting kit-8

MORF4L2 was associated with DNA repair and immunosuppression (Figure S2A, B). MORF4L2 was shown to be significantly positively correlated with the expression of RAD51, a core molecule for homologous recombination repair, which participates in DNA repair together with BRCA1/2 (Figure S2C). Immunoblotting assays confirmed that MORF4L2 participates in DNA double-strand repair via RAD51 (Figure S2D). To identify the relationship between MORF4L2 and the immune cells, tissues were collected from 6 patients for immunohistochemical (IHC) staining. According to our results, anti-PD1 therapy was less effective among patients with high MORF4L2 expression (Fig. 3A). To investigate the relationship between MORF4L2 expression and immune cells, multiple immunofluorescence (mIF) analysis was carried out. The results suggested that a lower infiltration level of CD8 + T cells and a higher infiltration level of M2 type macrophages were observed in patients with high MORF4L2 expression (Fig. 3B). In addition, MORF4L2 mRNA expression was negatively correlated with PDCD1 expression (Figure S2C). Based on TCGA and FUSCC databases, MORF4L2 mRNA expression was dramatically elevated in tumor tissues compared with non-carcinoma samples (Fig. 3C). Furthermore, MORF4L2 was subsequently verified to be highly expressed in tumor cells, especially in metastatic tumor cells (Fig. 3D, E and Figure S2D). As discovered by collecting follow-up data, patients with high MORF4L2 expression had shorter progression-free survival (PFS) time (Fig. 3F, G). Additionally, the function of MORF4L2 in TNBC was explored. Based on MORF4L2 expression within different cells (Figure S3A), the MORF4L2 knockdown cell lines were constructed in human (SUM159, MDA-MB-231) and mouse (4T1) TNBC cells (Fig. 3H, I and Figure S3B-E). Through cell counting kit-8 and clonogenic assays, MORF4L2 showed no significant effect on tumor proliferation in vitro (Fig. 3J, K and Figure S3F, H). Thus, MORF4L2 is highly expressed in TNBC and might induce immune escape by modulating the immune microenvironment.

### MORF4L2 triggers macrophage recruitment and M2 polarization

**Fig. 4:**
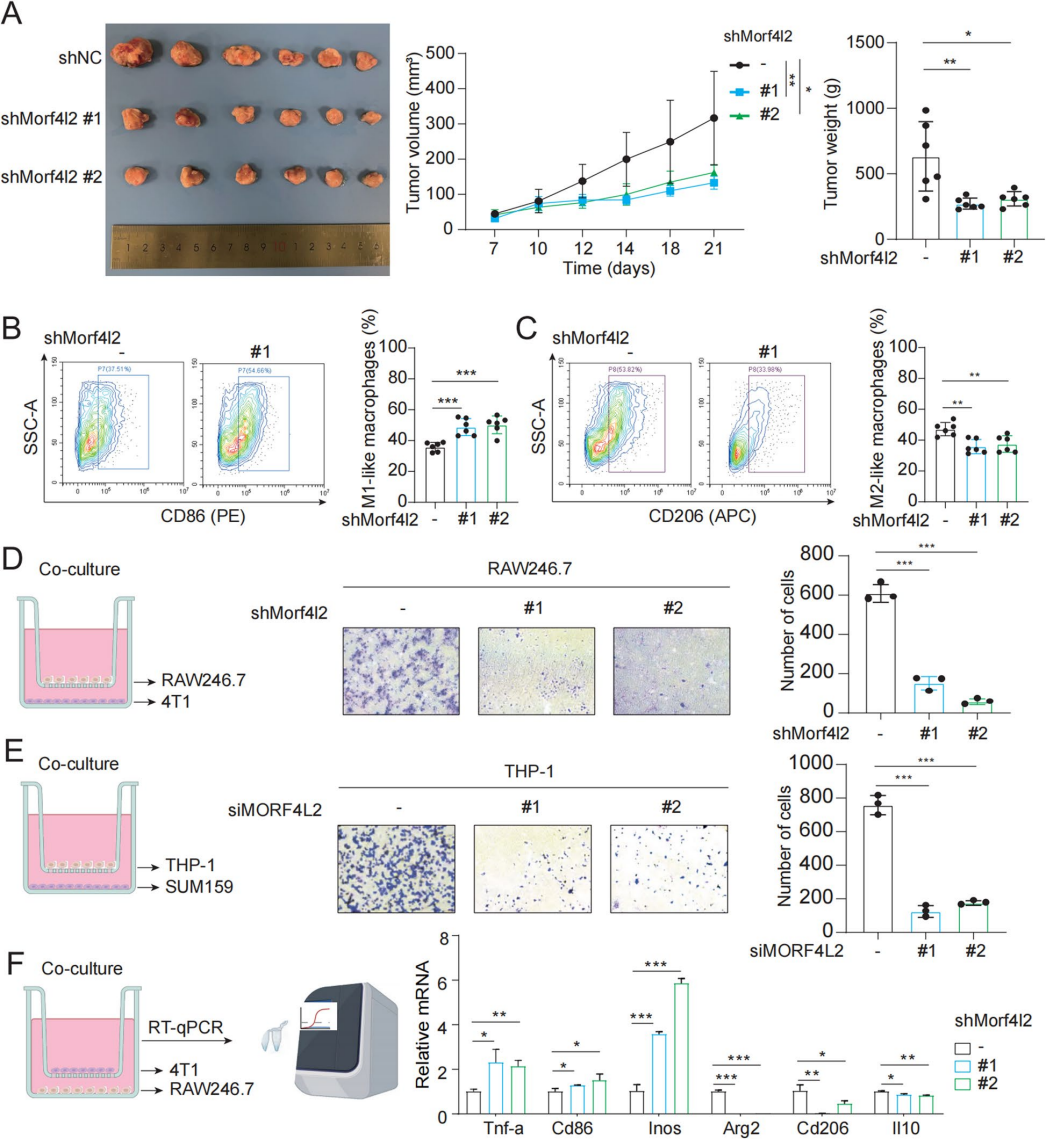
MORF4L2 induces macrophage recruitment and M2 polarization. **A** TNBC cells were injected into BALB/c mice, tumor volume was measured at regular intervals and tumor weight was measured at the end point. **B**, **C** Flow cytometry detected the expression of CD86 and CD206 in tumor tissues. **D**, **E** Macrophage chemotaxis and migration assay (**F**) RT-qPCR detection of Tnf-a, Cd86, Inos, Arg2, Cd206, Il10 mRNA levels in macrophages. Data are presented as mean ± SD; *P < 0.05, **P < 0.01, ***P < 0.001, ns no significance. MORF4L2 mortality factor 4 like 2; TNBC triple-negative breast cancer

The xenograft tumor models were constructed with BALB/c and C57BL/6 mice using 4T1 and AT3 cell lines, respectively. Tumors in the Morf4l2 knockdown group were significantly smaller and weighed less (Fig. 4 A and Figure S4A). Flow cytometry analysis of mouse tumors revealed that Morf4l2 was negatively related to M1 phenotype macrophages and positively related to M2 phenotype macrophages (Fig. 4B, C, Figure S4B, C, and Figure S5A). We speculated that MORF4L2 may induce immunosuppression by promoting the polarization and recruitment of macrophages. Therefore, a macrophage-tumor cell co-culture model was constructed, and it was verified that Morf4l2 induced the recruitment of mouse macrophage (RAW246.7) cells (Fig. 4D). In addition, photomacroglycerol-12-myristate-13-acetate (PMA) was used to promote THP-1 differentiation into macrophages before co-culture with SUM159 cells, and it was found that MORF4L2 promoted the recruitment of human macrophages (Fig. 4E). For macrophage polarization, Tnf-a, Cd86, and Inos mRNA levels were markedly up-regulated in macrophages co-cultured with the Morf4l2 knockdown group, whereas those of Arg2, Cd206, and Il10 were significantly down-regulated (Fig. 4F). Together, MORF4L2 was confirmed to induce an immunosuppressive microenvironment by affecting macrophages through in vivo and in vitro experiments.

### MORF4L2, a component of NuA4 HAT complex, promotes macrophage polarization and recruitment through activating CSF1 transcription

**Fig. 5:**
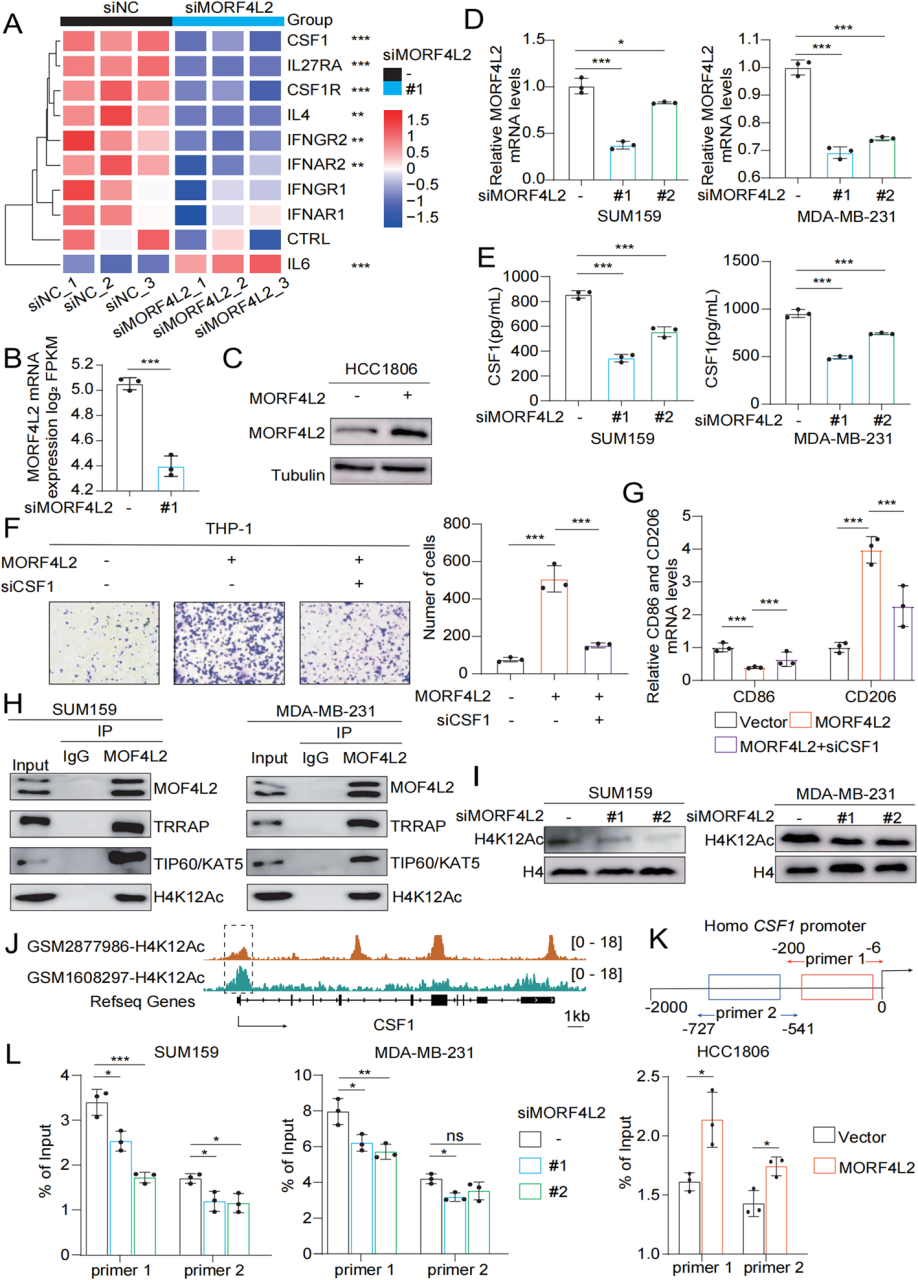
MORF4L2 promotes CSF1 transcription and regulates macrophages by constituting NuA4 HAT complex. **A** Critical gene analysis by RNA sequencing in control and MORF4L2 knockdown group. **B** RNA sequencing revealed downregulation of CSF1 mRNA expression after MORF4L2 knockdown. **C** Overexpression efficiency of MORF4L2 was analyzed using immunoblotting. **D**, **E** Detection of CSF1 expression by RT-qPCR and ELISA. **F** Macrophage chemotactic migration assays were performed on the control, MORF4L2 and MORF4L2 + siCSF1 groups. **G** RT-qPCR detection of CD86 and CD206 mRNA levels in macrophages. **H** Immunoprecipitation and Western blot analyses were performed to verify the interaction between MORF4L2, TIP60/KAT5, and TRRAP. **I** Immunoblotting identifies histone H4K12 acetylation modifications. **J** The visualization of enrichment of H4K12Ac at CSF1 gene locus. Both of data were normalized using reads per genomic content (RPGC). The peak at transcript start site of CSF1 were enclosed with dash line. **K**, **L** ChIP-qPCR analysis of H4K12Ac occupancy of the CSF1 promoter region in cells knocking down or overexpressing MORF4L2. Data are presented as mean ± SD; *P < 0.05, **P < 0.01, ***P < 0.001, ns no significance. CSF1 colony stimulating factor; MORF4L2 mortality factor 4 like 2; H4K12Ac histone 4 lysine 12 acetylation; ELISA eznyme linked immunosorbent assay; TIP60/KAT5 lysine acetyltransferase 5; TRRAP transformation/transcription domain-associated protein; ChIP chromatin immunoprecipitation

To further clarify the downstream mechanism by which MORF4L2 regulates immune escape in TNBC, RNA sequencing was conducted on SUM159 cells (Fig. 5A, B). The results suggested that CSF1 mRNA expression markedly decreased in MORF4L2 knockdown group (Fig. 5D). Subsequently, ELISA assay verified that the CSF1 expression was lower in the supernatants of MORF4L2 knockdown cells than in those of control cells (Fig. 5E). Moreover, knockdown of CFS1 partially reversed the effects on macrophage recruitment and M2 polarization effects induced by MORF4L2 overexpression in HCC1806 cells (Fig. 5C, F, G). Therefore, CSF1 was inferred to be an important transcriptional target. Previous studies have suggested that MORF4L2 affects histone 4 acetylation as part of the NuA4 HAT complex. Co-IP assay confirmed that MORF4L2 together with several proteins such as lysine acetyltransferase 5 (TIP60/KAT5), and transformation/transcription domain-associated protein (TRRAP), etc. constitute the NuA4 HAT complex (Fig. 5H). Thereafter, immunoblotting analysis was carried out, which confirmed that H4K12Ac was significantly down-regulated after MORF4L2 knockdown (Fig. 5I). In addition, ChIP sequencing from GEO database was used, revealing significant enrichment of CSF1 gene locus H4K12Ac (Fig. 5J). ChIP-qPCR assay showed that MORF4L2 increased H4k12Ac enrichment of the CSF1 promoter region in TNBC cells (Fig. 5K, L). These results suggested that MORF4L2 activated CSF1 transcription to enhance macrophage recruitment and M2 polarization mainly through H4K12Ac.

### MORF4L2 is transcriptionally regulated by GRHL2

**Fig. 6:**
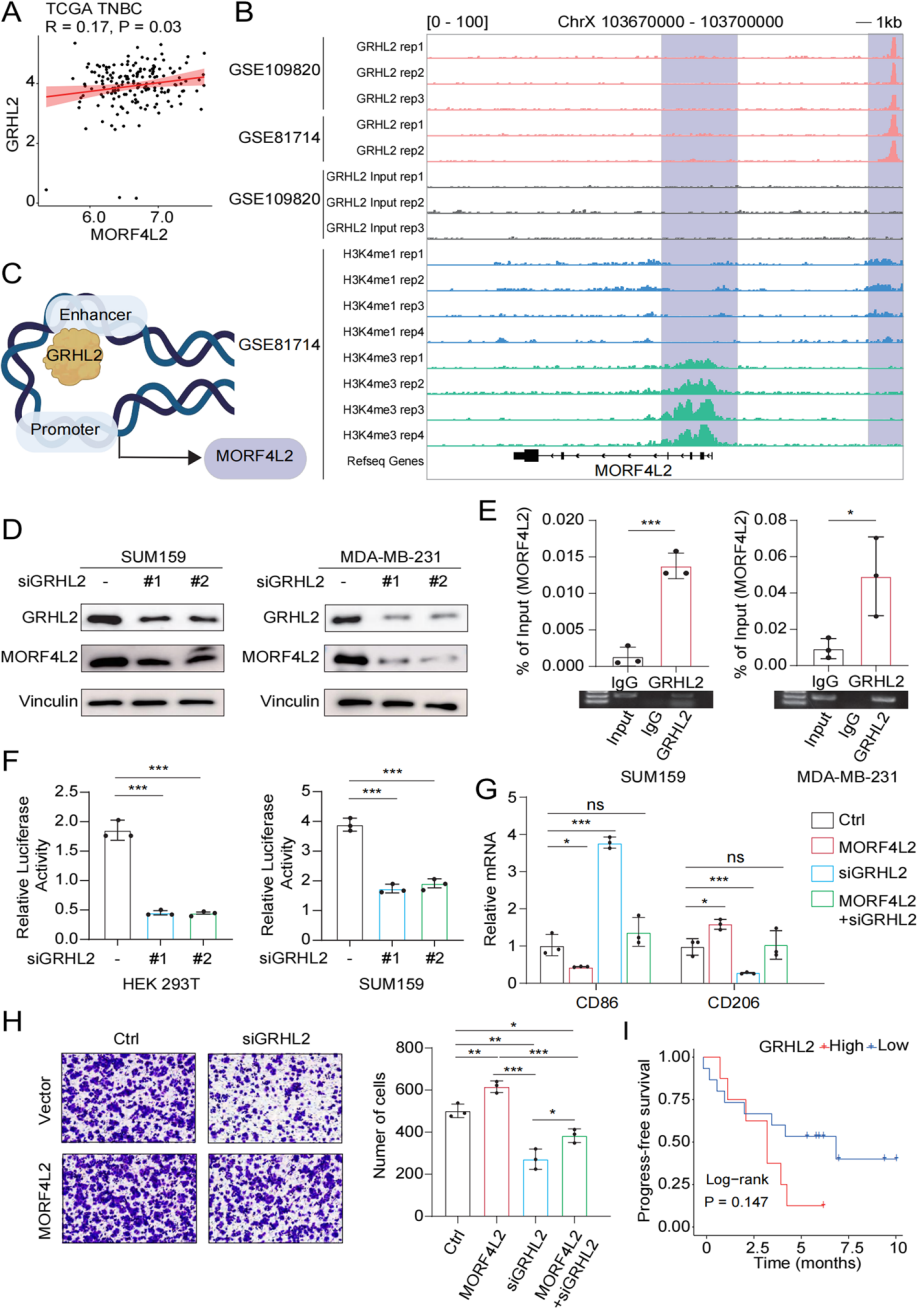
GRHL2 regulates transcription of MORF4L2. **A** Correlation analysis of MORF4L2 and GRHL2 expression in the TCGA TNBC transcriptome databases. **B** ChIP-seq peaks of GRHL2, H3K4me1 and H3K4me3 in the MORF4L2 enhancer region. **C** Schematic representation of the molecular mechanism by which GRHL2 drives MORF4L2 transcription. **D** Protein expression levels of GRHL2 and MORF4L2 after knockdown of GRHL2. **E** ChIP assay with GRHL2 antibody followed by RT-qPCR for MORF4L2 enhancer. **F** Detection and analysis of luciferase activity in HEK293T and SUM159 co-transfected with GRHL2 and pGL3-MORF4L2 enhancer plasmids. **G** RT-qPCR detection of CD86 and CD206 mRNA levels in macrophages. **H** Macrophage chemotactic migration assays were performed on the control, siGRHL2, MORF4L2 and MORF4L2 + siGRHL2 groups. **I** PFS curve based on GRHL2 expression was analyzed by log-rank test using the Kaplan–Meier method. Data are presented as mean ± SD; *P < 0.05, **P < 0.01, ***P < 0.001, ns no significance. MORF4L2 mortality factor 4 like 2; TNBC triple-negative breast cancer; GRHL2 grainyhead like transcription factor 2; PFS progression-free survival; ChIP chromatin immunoprecipitation

To elucidate the molecular mechanisms underlying the overexpression of MORF4L2 in TNBC, we first analyzed transcriptome data and found a positive correlation between the RNA expression levels of MORF4L2 and GRHL2 (Fig. 6A, Figure S5A). The role of GRHL2 in breast cancer is ambiguous, with previous studies showing both pro- and anti-metastatic effects [32, 33]. To verify the potential regulation to MORF4L2 transcription, we analyzed the ChIP sequencing data of GRHL2 and active histone modification marks (H3K4me1 and H3K4me3) at the MORF4L2 gene locus. Interestingly, we found that GRHL2 was enriched in the MORF4L2 enhancer region (Fig. 6B, C). Subsequently, we knocked down GRHL2 (Figure S5B) and observed a decrease in the mRNA and protein levels of MORF4L2 (Figure S5C and Fig. 6D). ChIP-qPCR assay confirmed the presence of GRHL2 binding sites in the MORF4L2 enhancer sequence (Fig. 6E). Then, the MORF4L2 enhancer region sequence was constructed into a pGL3 reporter plasmid, and HEK293T cells and SUM159 cells were transfected for dual luciferase reporter gene detection. The results showed that co-transfection of the GRHL2 overexpression plasmid with the MORF4L2 enhancer significantly increased luciferase activity (Fig. 6F). In addition, when MORF4L2 was overexpressed in GRHL2 knockdown cells, the GRHL2 deletion-induced effects on macrophage recruitment and M2 polarization were significantly alleviated (Fig. 6G, H). We wondered whether GRHL2 would also adversely affect the prognosis of TNBC patients receiving immunotherapy. Unsurprisingly, although statistical significance was not reached, we still observed that patients with high GRHL2 mRNA expression had a poorer prognosis in SPARK cohort (Fig. 6I). These results indicate that GRHL2 influences the interaction between tumor cells and macrophages by binding to the MORF4L2 enhancer region.

### BLZ945 improves the efficacy of anti-PD1 therapy via GRHL2/MORF4L2/H4K12Ac/CSF1 axis

**Fig. 7:**
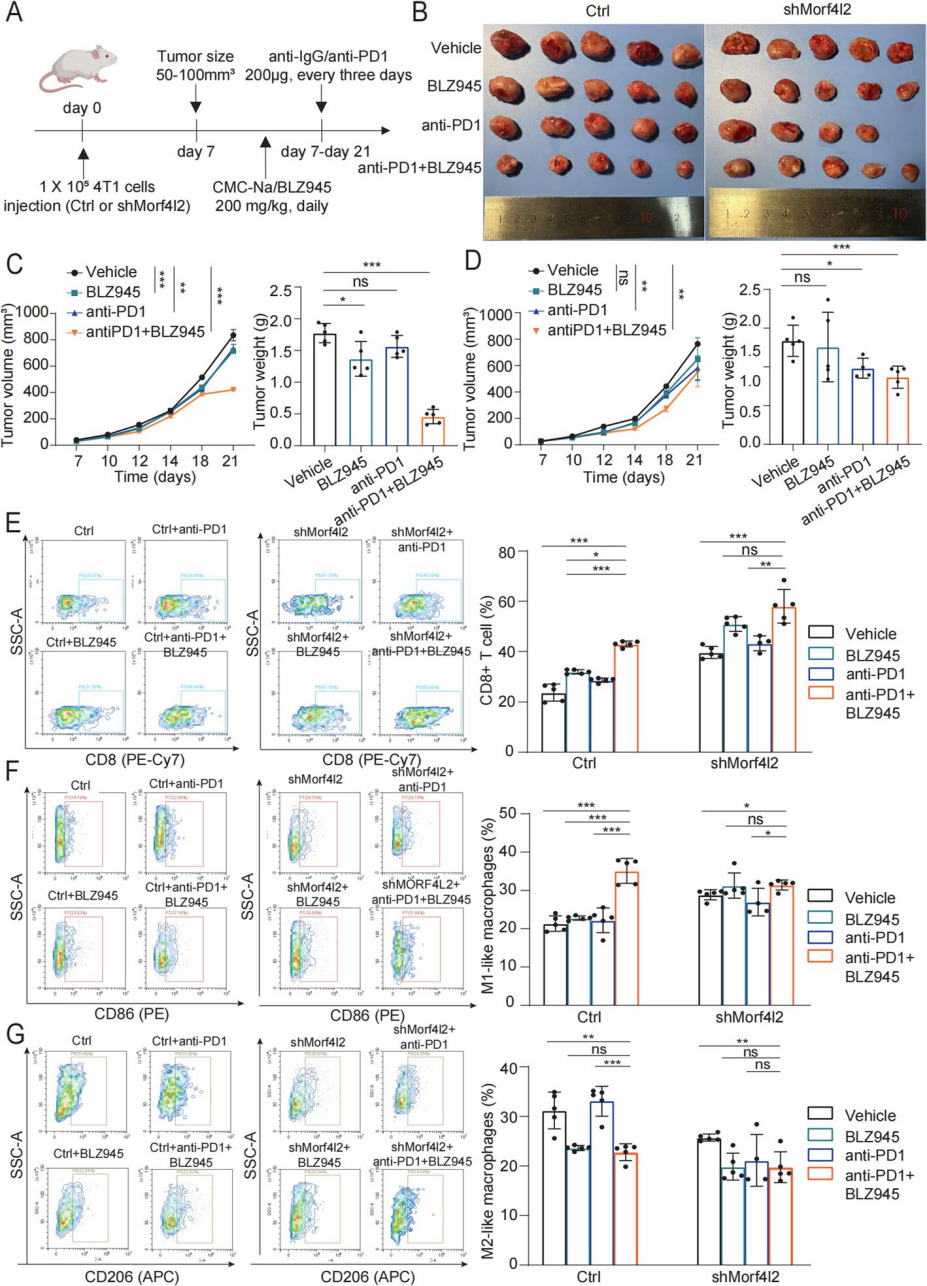
BLZ945 improves anti-PD1 efficacy by inhibiting the GRHL2/MORF4L2/H4K12A/CSF1 axis. Study design for each treatment group. **B**-**D** 4T1 cells were injected into BALB/c mice. Mice were treated with anti-PD1, BLZ945 or combination therapy. Tumor volume and weight were measured at the indicated times. **E**–**G** Flow cytometric analysis of microenvironmental cells in tumor tissues. Data are presented as mean ± SD; *P < 0.05, **P < 0.01, ***P < 0.001, ns no significance. MORF4L2 mortality factor 4 like 2; GRHL2 grainyhead like transcription factor 2; CSF1 colony stimulating factor; H4K12Ac histone 4 lysine 12 acetylation

**Fig. 8:**
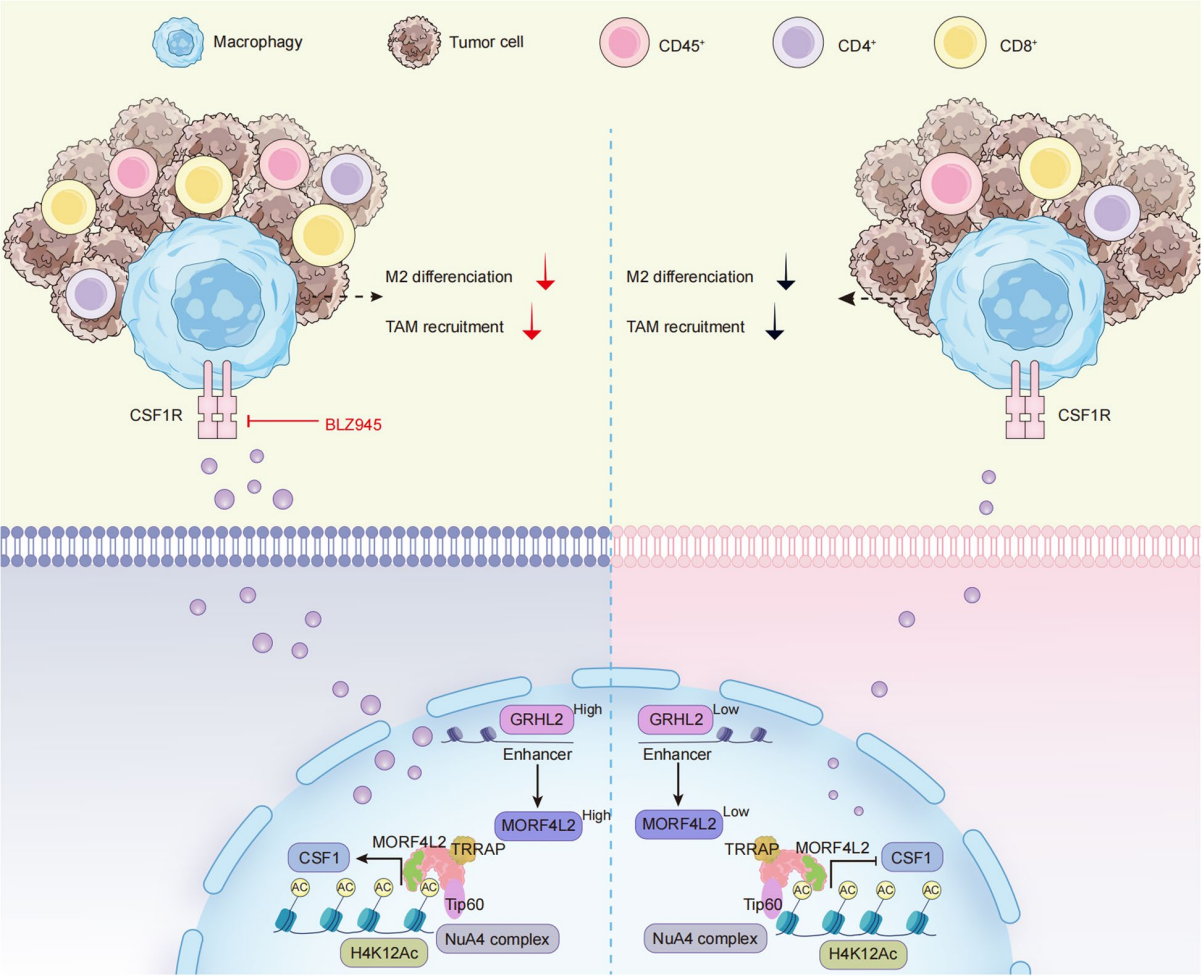
The model demonstrates the multifaceted role of MORF4L2 in breast cancer-macrophage communication, macrophage recruitment and polarization. Data are presented as mean ± SD; *P < 0.05, **P < 0.01, ***P < 0.001, ns no significance

BLZ945, a targeted CSF1R inhibitor, was utilized to block the MORF4L2/H4K12Ac/CSF1 axis. In the Morf4l2 knockdown group, anti-PD1, BLZ945, and BLZ945 plus anti-PD1 partially suppressed tumor growth, whereas in the control group, BLZ945 plus anti-PD1 evidently suppressed tumor growth (Fig. 7A-D). Thus, the combination therapy significantly improved the anti-PD-1 efficacy in Morf4l2 over-expression mice. Flow cytometry analysis revealed the increased numbers of CD8 + T cells and CD86 + macrophages (M1-like macrophages) as well as the significantly decreased numbers of CD206 + macrophages (M2-like macrophages) in the combination therapy group compared with those in the control group (Fig. 7E-G and Figure S5A, B). Therefore, MORF4L2 could be a candidate biomarker for the combined use of BLZ945 and anti-PD1 (Fig. 8).

## Discussion

Anti-PD(L)1 therapy, represented by camrelizumab and atezolizumab, has been a major success in the treatment of advanced TNBC [26, 34, 35]. Although immunotherapy can significantly improve survival time, only a fraction of patients with TNBC can benefit from immunotherapy. For instance, in the IMpassion 130 clinical trial, researchers reported that immunotherapy was more effective in PD-L1-positive patients than in their PD-L1-negative counterparts, but only 40.9% of patients were PD-L1 positive. Additionally, FUTURE-SUPER clinical trial defined CD8 positivity as an immunomodulatory subtype, and thus immunotherapy was administered to these patients, yet CD8-positive patients only 42.4% of the total patients. Owing to the limited efficacy of anti-PD1 therapy in TNBC patients, more novel targets for sensitizing patients to anti-PD1 therapy or biomarkers for predicting treatment efficacy are urgently needed. In this study, we propose for the first time that MORF4L2 is positively associated with an unfavorable response to immunotherapy in TNBC patients. Specifically, MORF4L2, as part of the NuA4 HAT complex, acetylates the activated H4K12, which in turn promotes the transcription of CSF1, a molecule that acts directly on CSF1R on the macrophage surface. GRHL2, as a transcription factor, can upstreamly regulate the transcription of MORF4L2. Clinically, a promising immunotherapy combination regimen, namely, an anti-PD1 plus CSF1R inhibitor (BLZ945), is designed to target the GRHL2/MORF4L2/H4K12Ac/CSF1 axis.

Several studies have demonstrated that MORF4L2 is related to a variety of diseases and immunosuppression [21, 36–38]. MORF4L2, which is part of NuA4 HAT complex, relates to transcription activation of selected genes, mainly by acetylating nucleosome histones H4 and H2A [39, 40]. Histone acetylation is a chromatin modification controlled by HATs and histone deacetylase (HDAC) complex [41]. Acetylation negatively charges lysine residues in the N-terminal histone tails protruding from the nucleosome, and these negative charges repel negatively charged DNA, resulting in a relaxed chromatin structure and an open chromatin conformation that permits the binding of transcription factors and a significant increase in gene expression. NuA4 HAT, a 12-subunit complex first identified in yeast, is primarily responsible for the acetylation of histone H4 in yeast and is structurally and functionally highly conserved in both humans and yeast [42]. Its catalytic component, Esa1 (the human homologue as lysine acetyltransferase 5 (TIP60/KAT5)), is involved in RNA transcription, circadian rhythms, DNA repair, and tumor progression [43–45]. Other subunits include Tra1 (human homologue as transformation/transcription domain-associated protein (TRRAP)), an ATM family cofactor associated with the recruitment of transcriptional activators [46]. In addition, NuA4 HAT complex also includes Yng2 (human homologue as inhibitor Of growth family member 3 (ING3)), Arp4 (human homologue as BRG1-associated factor 53a (BAF53A)), Epl1 (human homologue as enhancer of polycomb homolog 1 (EPC1)) and others [43]. Moreover, MORF4L2 is involved in DNA homologous recombination repair via its interaction with the DNA repair factor called partner and localizer of BRCA2 (PALB2) [22]. It has been demonstrated that MORF4L2, a DNA repair-related gene, promotes MCF7 cell proliferation and facilitates macrophage and CD8 + T cell infiltration [21]. Our assays also confirmed that MORF4L2 can regulate DNA double-strand repair via RAD51. Since it has been reported that TIP60 can be recruited to DNA-damaged regions, we speculate that this process is likely to be dependent on the NuA4 HAT complex [47]. However, the relationship between MORF4L2 and the immune microenvironment has not been fully elucidated. Only one study on head and neck squamous cell carcinoma found that MORF4L2 was a prognostic biomarker for immunotherapy [37]. Through the screening of sequencing data from SPARK cohorts, we revealed that MORF4L2 may be significantly associated with immunotherapy resistance, but the exact mechanism by which it regulates immunity remains unclear.

This study is the first to demonstrate that MORF4L2 induces the generation of an immunosuppressive microenvironment in TNBC. Through RNA sequencing, we focused on CSF1, a cytokine secreted by tumor cells, that is important for promoting macrophage recruitment and polarization [48, 49]. For direct mechanistic studies, immunoblotting assays and genomic sequencing data analysis were performed, which confirmed that MORF4L2 regulated CSF1 transcription by acetylating histones. Binding of transcription factors to enhancers is a crucial step in transcriptional activation and one of the common characteristics of cancer. Previous studies have shown that H3K4me1 characterizes the enhancer region and that H3K4me3 is enriched at the promoters of the encoded genes [50]. Our study revealed that H3K4me1 on enhancers was enriched at GRHL2-bound enhancer elements. ChIP sequencing combined with ChIP-qPCR and luciferase was also used to verify our hypotheses [51–53]. Taken together, this study underscores the importance of the GRHL2/MORF4L2/H4K12Ac/CSF1 axis in promoting the immunosuppressive microenvironment and immunotherapy resistance.

CSF1R can be used to treat chronic graft-versus-host disease and tumors [54, 55]. As shown by several preclinical and clinical studies, CSF1R in combination with anti-PD1 significantly improves the objective remission rates in patients with solid tumors such as glioma, melanoma, urinary bladder cancer, and non-small cell lung cancer [56, 57]. With regard to breast cancer, mouse experiments have suggested that CSF1R inhibitors alone or in combination with chemotherapy can be used to treat TNBC [58, 59]. Therefore, a CSF1R inhibitor (BLZ945) in combination with anti-PD1 was selected. Our results further support that BLZ945 combined with anti-PD1 enhances the efficacy of immunotherapy, particularly in mice with high MORF4L2 expression.

This study possesses certain limitations. Firstly, we only collected patient tissues from the SPARK cohort, which is not representative of characteristics of all the advanced TNBC patients, and large-scale histological data from patients receiving immunotherapy are needed to validate our findings. Secondly, H4K12 may be only one of the MORF4L2 acetylation sites, and the effect of MORF4L2 on histone complexes remains to be further elucidated.

In conclusion, we identify MORF4L2, the predictive biomarker for immunotherapy efficacy in advanced TNBC patients, in this study. Our results demonstrate that the GRHL2/MORF4L2/H4K12Ac/CSF1 axis promotes the resistance to anti-PD1 therapy by recruiting macrophages and promoting their M2 polarization. And, BLZ945, an inhibitor targeting CSF1R, reverses the anti-PD1 resistance. This study reveals a novel strategy for TNBC immunotherapy and provides a basis for the precise treatment of TNBC.

## Supplementary Information

Supplementary Material 1.

## Data Availability

The data that support the findings of this study are available from the corresponding author upon reasonable request.

## Abbreviations

MORF4L2: Mortality factor 4 like 2
TNBC: Triple-negative breast cancer
PD1: Programmed cell death protein-1
CSF1: Colony stimulating factor 1
PD-L1: Programmed cell death-ligand 1
HAT: Histone acetyltransferase
H4K12Ac: Histone 4 lysine 12 acetylation
CSF1R: CSF1 receptor
TAMs: Tumor-associated macrophages
PALB2: Partner and localizer of BRCA2
PD: Disease progression
CR: Complete response
PR: Partial response
SD: Stable disease
DCR: Disease control rate
DEG: Differentially expressed gene
GSEA: Gene set enrichment analysis
scRNA-seq: Single cell RNA sequencing
PARP1: Poly (ADP-ribose) polymerase 1
NME1: NME/NM23 nucleoside diphosphate kinase 1
PCNA: Proliferating cell nuclear antigen
UMAP: Uniform manifold approximation and projection
GSVA: Genomic variation analysis
MsigDB: Molecular Signatures Database
IHC: Immunohistochemical
mIF: Multiple immunofluorescence
TCGA: The Cancer Genome Atlas
FUSCC: Fudan University Shanghai Cancer Center
PMA: Photomacroglycerol-12-myristate-13-acetate
ChIP: Chromatin immunoprecipitation
GEO: Gene Expression Omnibus
RECIST: Response Evaluation Criteria in Solid Tumors
ssGSEA: Single sample gene set enrichment analysis
ECL: Electrogenerated chemiluminescence
HRP: Horseradish peroxidase
DAB: 3,3'-Diaminobenzidine
CCA: Canonical correlation analysis
GRHL2: Grainyhead like transcription factor 2
TIP60/KAT5: Lysine acetyltransferase 5
TRRAP: Transformation/transcription domain-associated protein
ING3: Inhibitor of growth family member 3
BAF53A: BRG1-associated factor 53a
EPC1: Enhancer of polycomb homolog 1
Co-IP: Coimmunoprecipitation
