# Correction: MORF4L2 induces immunosuppressive microenvironment and immunotherapy resistance through GRHL2/MORF4L2/H4K12Ac/CSF1 axis in triple-negative breast cancer

**DOI:** 10.1186/s40364-025-00743-9

**Published:** 2025-02-13

**Authors:** Xin-Yi Sui, Shuo-Wen Cao, Xiao-Qing Song, Xi-Yu Liu, Chao Chen, Qingya Yan, Zhi-Qing Wang, Wen-Juan Zhang, Lin-Xiaoxi Ma, Xi Jin, Ding Ma, Yi Xiao, Song-Yang Wu, Ying Xu, Zhi-Ming Shao, Lei Fan

**Affiliations:** 1 Department of Breast Surgery, Fudan University Shanghai Cancer Center, Shanghai, China; 2 Key Laboratory of Breast Cancer in Shanghai, Department of Oncology, Shanghai Medical College, Fudan University, Shanghai, China; 3 School of Basic Medical Sciences, Xinxiang Medical University, Xinxiang, China

**Correction: Biomarker Research (2025) 13:6**

10.1186/s40364-024-00719-1

The authors wish to note that in the sentence, “Notably, BLZ549, an inhibitor of the CSF1R [...]” in the Abstract, ‘BLZ549’ should instead state ‘BLZ945’.

