# Supplementary material for: MORF4L2 induces immunosuppressive microenvironment and immunotherapy resistance through GRHL2/MORF4L2/H4K12Ac/CSF1 axis in triple-negative breast cancer
Source: Biomark Res. 2025 Jan 9;13:6. doi: 10.1186/s40364-024-00719-1 (PMC11715975; doi:10.1186/s40364-024-00719-1)
Supplement: Supplementary file 1 — Supplementary Material 1. [file 40364_2024_719_MOESM1_ESM.pdf]

Supplementary Materials

MORF4L2 Induces Immunosuppressive Microenvironment and Immunotherapy Resistance through GRHL2/MORF4L2/H4K12Ac/CSF1 Axis in Triple-Negative Breast Cancer

Authors

Xin-Yi Sui [1,2†], Shuo-Wen Cao [1,2†], Xiao-Qing Song [1,2†], Xi-Yu Liu [1,2†], Chao Chen [1,2], Qingya Yan [3], Zhi-Qing Wang [1,2], Wen-Juan Zhang [1,2], Lin-Xiaoxi Ma [1,2], Xi Jin [1,2], Ding Ma [1,2], Yi Xiao [1,2], Song-Yang Wu [1,2], Ying Xu [1,2], Zhi-Ming Shao [1,2*], Lei Fan [1,2*]

Affiliations

[1]Department of Breast Surgery, Fudan University Shanghai Cancer Center, Shanghai, China

[2]Key Laboratory of Breast Cancer in Shanghai, Department of Oncology, Shanghai Medical College, Fudan University, Shanghai, China

[3]School of Basic Medical Sciences, Xinxiang Medical University, Xinxiang, China.

*** Correspondence to**

Dr. Lei Fan, Fudan University Shanghai Cancer Center, 270 Dong-An Road, Shanghai 200032, P.R. China, Tel: 86-21-64175590, Fax: 86-21-64434556

Email: drfanlei@outlook.com

Dr. Zhi-Ming Shao, Fudan University Shanghai Cancer Center, 270 Dong-An Road, Shanghai 200032, P.R. China, Tel: 86-21-64175590, Fax: 86-21-64434556

Email: zhi_ming_shao@163.com

↑These authors contributed equally to this work and should be considered as co-first authors.

The file includes:

Figures S1 to S6

Tables S1 to S5

Supplementary figures

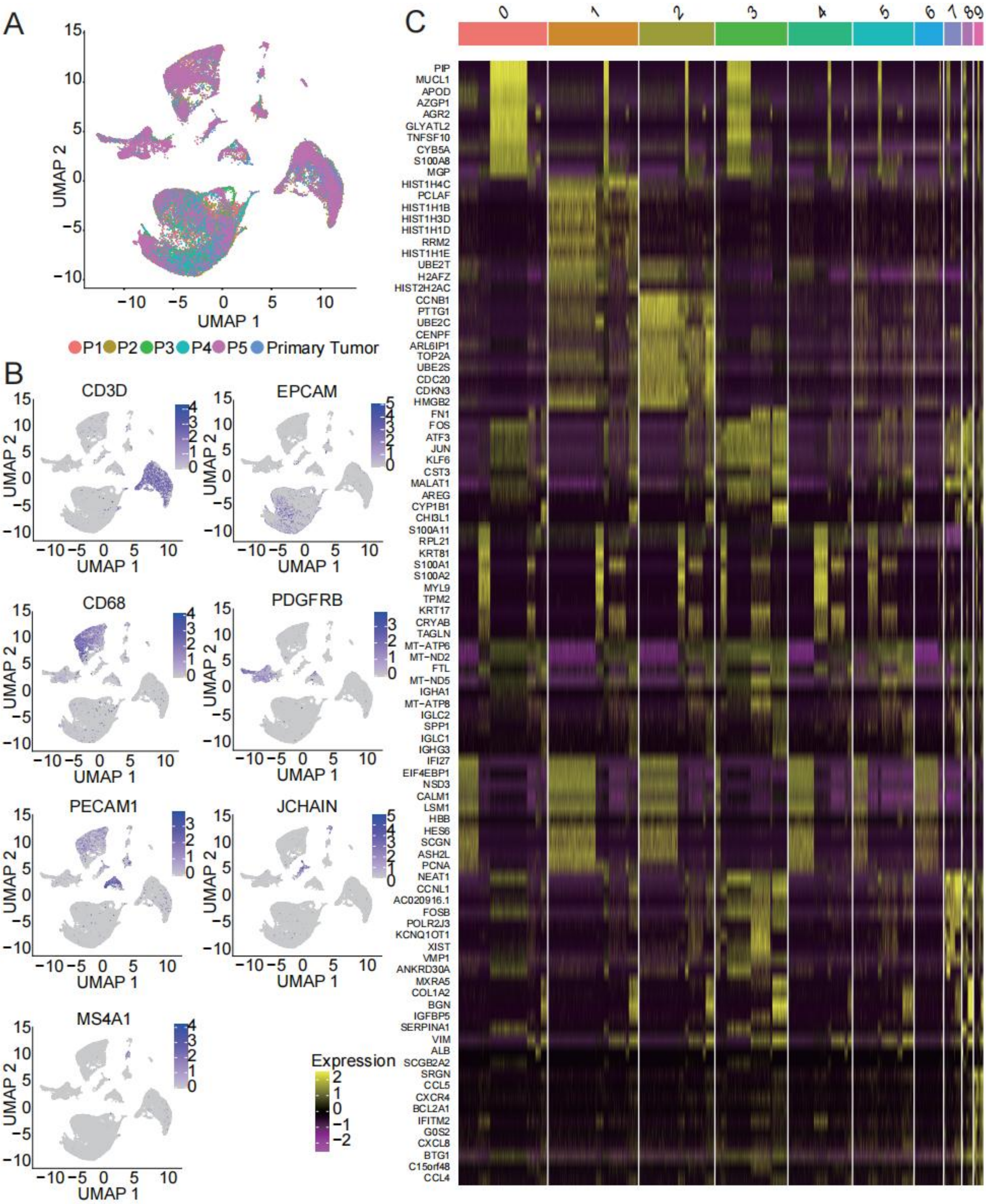

Figure S1 Clusters of patient microenvironmental cells and tumor cells.

(A) UMAP plots of metastatic tumor tissue from 5 breast cancer patients and 1 paired primary tumor, each patient displayed in a different color. (B) Dot plots of marker genes expressed in major cell types, with dot colors reflecting expression levels. (C) Top10 DEGs in different clusters.

UMAP uniform manifold approximation and projection; DEGs differentially expressed genes.

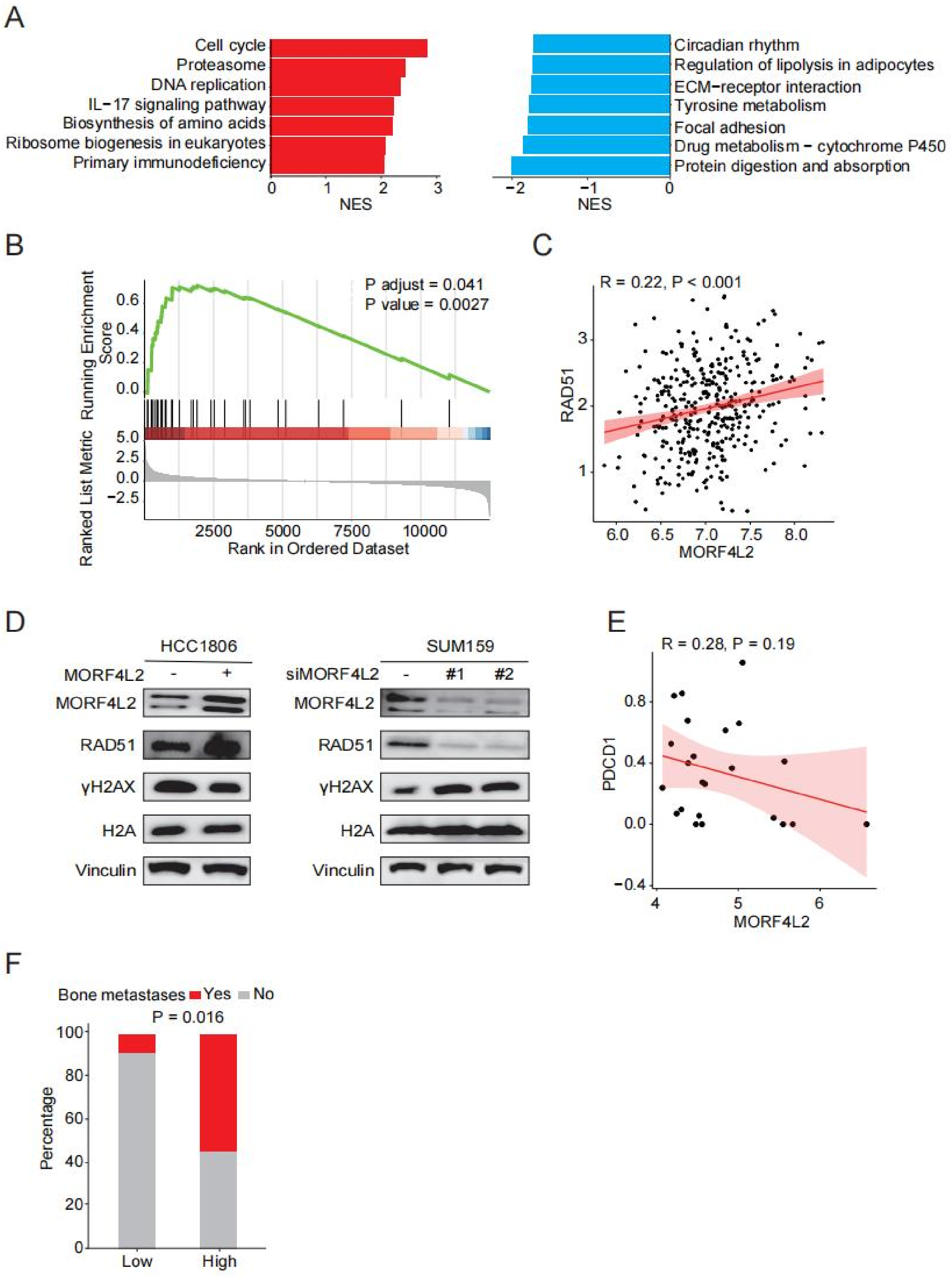

A
Cell cycle
Proteasome
DNA replication
IL−17 signaling pathway
Biosynthesis of amino acids
Ribosome biogenesis in eukaryotes
Primary immunodeficiency
NES
Circadian rhythm
Regulation of lipolysis in adipocytes
ECM−receptor interaction
Tyrosine metabolism
Focal adhesion
Drug metabolism − cytochrome P450
Protein digestion and absorption
NES
B
P adjust = 0.041
P value = 0.0027
Running Enrichment Score
Ranked List Metric
Rank in Ordered Dataset
C
R = 0.22, P < 0.001
RAD51
MORF4L2
D
HCC1806
MORF4L2 - +
SUM159
siMORF4L2 - #1 #2
MORF4L2
RAD51
γH2AX
H2A
Vinculin
E
R = 0.28, P = 0.19
PDCD1
MORF4L2
F
Bone metastases Yes No
P = 0.016
Percentage
Low
High

Figure S2 Relationship between MORF4L2 expression and clinical characteristics among TNBC patients.

(A, B) Enrichment analysis of the SPARK cohort grouped by MORF4L2 mRNA expression. (C) MORF4L2 was positively correlated with RAD51 mRNA expression. (D) MORF4L2, RAD51 and γ H2AX proteins content in HCC1806 and SUM159 cells treated with camptothecin for 4 hours. (E) MORF4L2 was negatively correlated with PDCD1 mRNA expression. (F) MORF4L2 was associated with bone metastasis in SPARK cohort.

MORF4L2 mortality factor 4 like 2; TNBC triple-negative breast cancer.

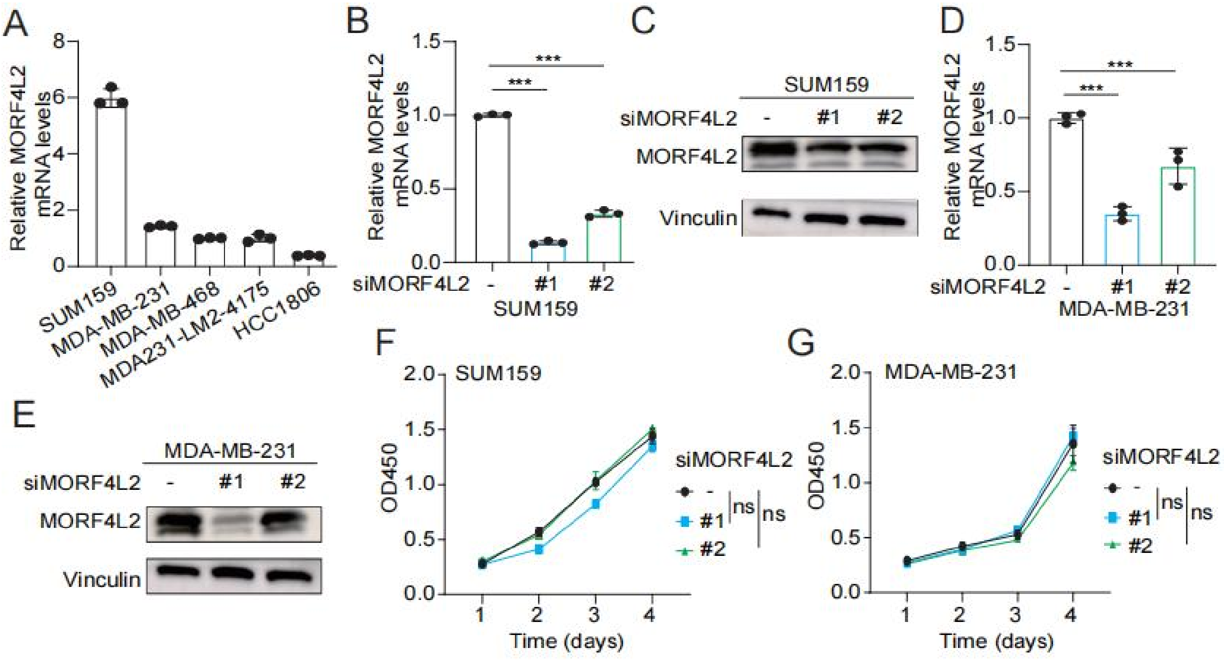

Figure S3 MORF4L2 failed to promote TNBC cell proliferation in vitro.

(A) Relative expression levels of MORF4L2 mRNA in different cells. (B-E) RT-qPCR and immunoblotting were used to analyze the knockdown efficiency of MORF4L2. (F, G) CCK8 was used to identify cell proliferation. Data are presented as mean ± SD; *P < 0.05, **P < 0.01, ***P < 0.001, ns no significance.

MORF4L2 mortality factor 4 like 2; TNBC triple-negative breast cancer.

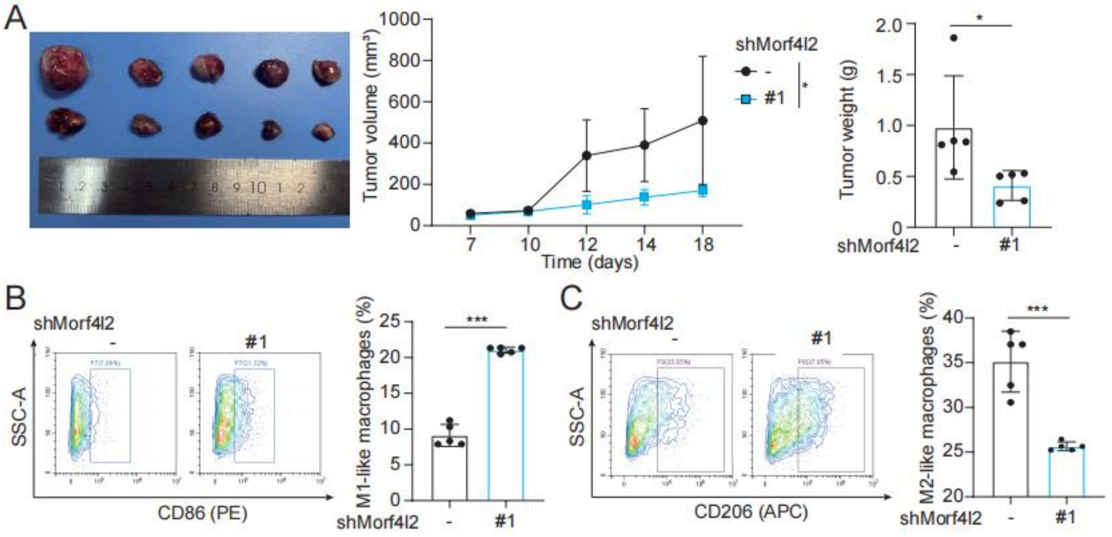

Figure S4 MORF4L2 promotes tumor growth in vivo by inducing macrophage recruitment and M2 polarization.

(A) AT3 cells were injected into C57BL/6 mice and tumor volume was measured at regular intervals and tumor weight at endpoint. (B, C) Flow cytometry to detect the expression of CD86 and CD206 in tumor tissues.

MORF4L2 mortality factor 4 like 2.

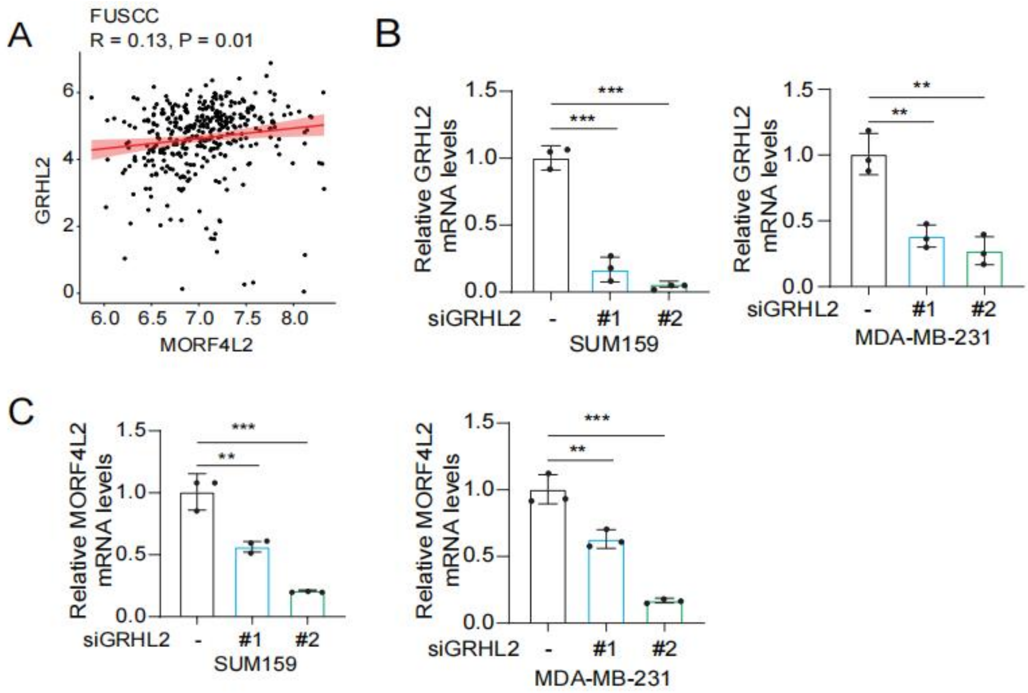

Figure S5 GRHL2 is positively correlated with MORF4L2 at the mRNA level.

(A) Correlation analysis of MORF4L2 and GRHL2 expression in the FUSCC transcriptome databases. (B) mRNA level of GRHL2 in SUM159 and MDA-MB-231 cells post GRHL2 siRNA transfection. (C) mRNA level of MORF4L2 in SUM159 and MDA-MB-231 cells post GRHL2 siRNA transfection.

MORF4L2 mortality factor 4 like 2; GRHL2 grainyhead like transcription factor 2.

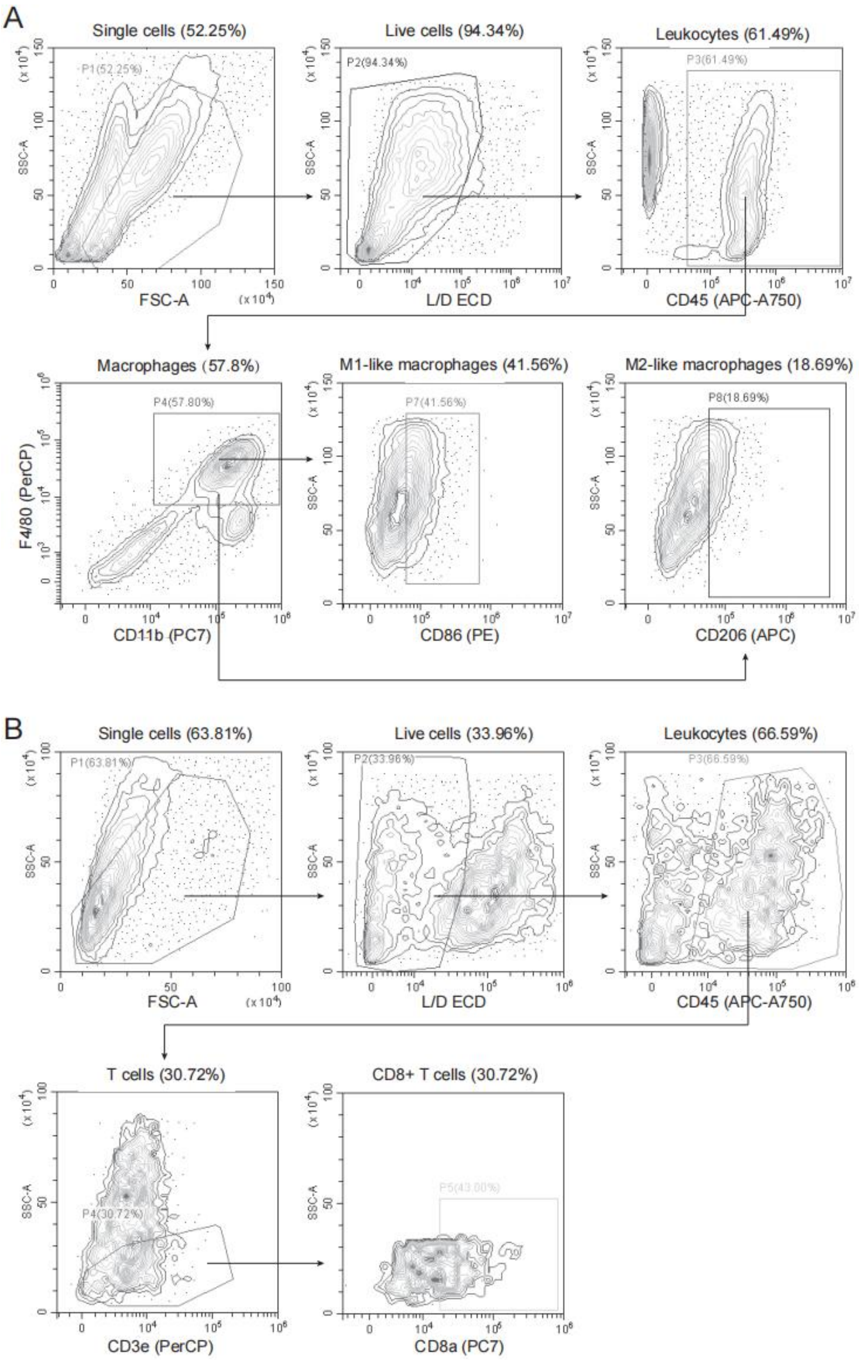

Figure S6 Gating strategy for flow cytometry analysis.

(A) Flow cytometry analysis of single cells (FSC-A+), live cells (L/D ECD-), leukocytes (CD45+), macrophages (CD45+CD11b+F4/80+), M1-like macrophages (CD45+CD11b+F4/80+CD86+), and M2-like macrophages (CD45+CD11b+F4/80+CD206+).

(B) Flow cytometry analysis of single cells (FSC-A+), live cells (L/D ECD-), leukocytes (CD45+), T cells (CD45+CD3e+), and CD8+ T cells (CD45+CD3e+CD8a+).

Supplementary tables

Table S1 Summary of target sequence

siMORF4L2-1	GCTCCAATGTCCCAGGTTT
siMORF4L2-2	GGAATATGCGGTTAATGAA
siCSF1	GATCCAGTGTGCTACCTTA
siGRHL2-1	GCCGATTACAAGGAGAGCTTT
siGRHL2-2	CCTTCAAAGCAGATGAAAGAA
shMorf4l2-1	CCTGAGATTATTCGTGAGAAT
shMorf4l2-2	CGTGGACAACAATCTGCTGAA

Table S2 Summary of primer sequence

MORF4L2	
Forward	5'-GACGGGTTCTCAACCTCGTG-3'
Reverse	5'-TGCATGTTGCTTCTAGTTGGTTT-3'
ACTB	
Forward	5'-CATGTACGTTGCTATCCAGGC-3'
Reverse	5'-CTCCTTAATGTCACGCACGAT-3'
Morf4l2	
Forward	5'-TGCCTCGGGAAAGAAGTCAG-3'
Reverse	5'-TCCTACTCTTGAATGCCTCCTC-3'
Actb	
Forward	5'-GTGACGTTGACATCCGTAAAGA-3'
Reverse	5'-GCCGGACTCATCGTACTCC-3'
Arg	
Forward	5'-CTCCAAGCCAAAGTCCTTAGAG-3'
Reverse	5'-AGGAGCTGTCATTAGGGACATC-3'
Cd206	
Forward	5'-CTCTGTTCAGCTATTGGACGC-3'
Reverse	5'-CGGAATTTCTGGGATTCAGCTTC-3'
Cd86	
Forward	5'-TGTTTCCGTGGAGACGCAAG-3'
Reverse	5'-TTGAGCCTTTGTAAATGGGCA-3'
Tnfa	
Forward	5'-CCCTCACACTCAGATCATCTTCT-3'
Reverse	5'-GCTACGACGTGGGCTACAG-3'
Nos2	
Forward	5'-GTTCTCAGCCCAACAATACAAGA-3'
Reverse	5'-GTGGACGGGTCGATGTCACG-3'
Il10	
Forward	5'-GCTCTTACTGACTGGCATGAG-3'
Reverse	5'-CGCAGCTCTAGGAGCATGTG-3'
CSF1	
Forward	5'-TGGCGAGCAGGAGTATCAC-3'
Reverse	5'-AGGTCTCCATCTGACTGTCAAT-3'
GRHL2	
Forward	5'-TCAATACCCGAAGAGCCTACA-3'
Reverse	5'-CTTGGCTGTCACTTGCTTTGC-3'
CD86	
Forward	5'-CTGCTCATCTATACACGGTTACC-3'
Reverse	5'-GGAAACGTCGTACAGTTCTGTG-3'
CD206	
Forward	5'-GGAGTGCCATCAAAAACGGA-3'
Reverse	5'-GTGTCATTTTTGCACTCCCAT-3'

Table S3 Summary of antibodies

Name	Supplier	Cat no.
Rabbit anti-CD68	servicebio	GB113150
Rabbit anti-CD206	servicebio	GB115273
Rabbit anti-CD8	servicebio	GB115692
Rabbit anti-MORF4L2	abcam	ab178405
Rabbit anti-TRRAP	abcam	ab183517
Rabbit anti-Tip60	abcam	ab300521
Mouse anti-Vinculin	proteintech	66305-1-Ig
Rabbit anti-Tubulin	proteintech	10094-1-AP
PE/Cyanine7 anti-mouse/human CD11b	BioLegend	101215
PE-Cy7 Rat Anti-Mouse CD8a	BD Biosciences	552877
PE anti-mouse CD86	BioLegend	105008
APC anti-mouse CD206	BioLegend	141708
PerCP/Cyanine5.5 anti-mouse CD3ε	BioLegend	100328
APC/Fire™ 750 anti-mouse CD45	BioLegend	103154
PerCP/Cyanine5.5 anti-mouse F4/80	BioLegend	123127
Rabbit anti-histone H4 (acetyl K12)	abcam	ab177793
Rabbit anti-histone H4	ThermoFisher	PA5-102611
Rabbit anti-GRHL2	SIGMA	HPA004820
Anti-Rabbit IgG (H+L)	Jackson ImmunoResearch	111-035-003
Anti-Mouse IgG (H+L)	Jackson ImmunoResearch	115-035-003

Table S4 Summary of primer sequence for CHIP-qPCR

CSF1 (CHIP-qPCR #1)	
Forward	5'-AAACCTGCTGACTCAGGCTC-3'
Reverse	5'-GCGCTCTATGATCGTCCCAA-3'
CSF1 (CHIP-qPCR #2)	
Forward	5'-TGTTTCTCCAGCACCAAGCA-3'
Reverse	5'-GATGTAACTGGCCCAGCAGT-3'
MORF4L2 (CHIP-qPCR)	
Forward	5'-AGTTCTTCCACAGCCCTCAGAT-3'
Reverse	5'-TGGAGGATAGGAAACCACACTGG-3'

Table S5 DNA damage repair genes

Gene Names							
AATF	CHD1L	FANCC	MDM4	POLH	RHNO1	TELO2	ZRANB3
ABL1	CHD4	FANCD2	MED17	POLI	RNASEH2A	TERF1	ZSWIM7
ACTR5	CHEK1	FANCE	MEIOB	POLK	RNF168	TERF2	
AKT1	CHEK2	FANCF	MEN1	POLL	RNF169	TERF2IP	
ALKBH1	CHRNA4	FANCG	MGME1	POLM	RNF8	TEX12	
ALKBH2	CIB1	FANCI	MGMT	POLN	RPA1	TEX15	
ALKBH3	CINP	FANCL	MLH1	POLQ	RPA2	TICRR	
AP5S1	CLSPN	FANCM	MLH3	POLR2A	RPA3	TMEM161A	
AP5Z1	COPS5	FBXO18	MMS19	POLR2B	RPA4	TNP1	
APEX1	CRB2	FBXO6	MMS22L	POLR2C	RPAIN	TONSL	
APEX2	CREB1	FEN1	MNAT1	POLR2D	RPS27A	TOP1	
APITD1	CREBBP	FGF10	MORF4L1	POLR2E	RPS27L	TOP2A	
APLF	CRY1	FHIT	MORF4L2	POLR2F	RPS3	TOP3A	
APTX	CRY2	FIGN	MPG	POLR2G	RRM2B	TOPBP1	
ASCC3	CSNK1D	FIGNL1	MRE11A	POLR2H	RTEL1	TP53	
ASF1A	CSNK1E	FOS	MSH2	POLR2I	RUVBL1	TP53BP1	
ASTE1	CUL4A	FOXM1	MSH3	POLR2J	RUVBL2	TP73	
ATF2	CUL4B	FTO	MSH4	POLR2K	SETD2	TREX1	
ATM	CYP19A1	FZR1	MSH5	POLR2L	SETMAR	TREX2	
ATMIN	CYP1A1	GADD45A	MSH6	PPM1D	SETX	TRIP12	
ATR	DAPK1	GADD45G	MTA1	PPP1CA	SFPQ	TRIP13	
ATRIP	DBF4	GEN1	MUM1	PPP2R2A	SFR1	TTC5	
ATRX	DCLRE1A	GPS1	MUS81	PPP2R5A	SHFM1	TWIST1	
ATXN3	DCLRE1B	GSTP1	MUTYH	PPP2R5B	SHPRH	TYMS	
AXIN2	DCLRE1C	GTF2H1	MYC	PPP2R5C	SIRT1	UBA1	
BABAM1	DDB1	GTF2H2	NABP1	PPP2R5D	SIRT6	UBA52	
BAP1	DDB2	GTF2H2C	NABP2	PPP2R5E	SLC30A9	UBB	
BARD1	DDR1	GTF2H3	NBN	PPP4C	SLX1A	UBC	
BAX	DDX1	GTF2H4	NCOA6	PPP4R2	SLX4	UBE2A	
BAZ1B	DEK	GTF2H5	NEIL1	PRKDC	SMAD2	UBE2B	
BCCIP	DHX9	H2AFX	NEIL2	PRMT6	SMAD3	UBE2D3	
BLM	DMAP1	HDAC1	NEIL3	PRPF19	SMAD4	UBE2I	
BRAP	DMC1	HDAC2	NEK1	PSMD3	SMAD7	UBE2N	
BRCA1	DNA2	HELQ	NEK11	PTTG1	SMARCA1	UBE2NL	
BRCA2	DOT1L	HERC2	NFKB1	RAD1	SMARCA2	UBE2T	
BRCC3	DTL	HIC1	NHEJ1	RAD17	SMARCA4	UBE2U	
BRE	DTX3L	HINFP	NINL	RAD18	SMARCA5	UBE2V2	
BRIP1	DUSP3	HIST3H2A	NME1	RAD21	SMARCAD1	UBE4B	
BTG2	DYRK2	HMGB1	NONO	RAD23A	SMARCB1	UHRF1	
BUB1	E2F1	HMGB2	NSMCE1	RAD23B	SMARCC2	UIMC1	
BUB1B	E2F2	HUS1	NSMCE2	RAD50	SMARCD1	UNG	
C11orf30	E2F4	HUS1B	NTHL1	RAD51	SMARCD2	UPF1	
C17orf70	E2F6	HUWE1	NUDT1	RAD51AP1	SMC1A	USP1	

C19orf40	EEPD1	IFI16	OGG1	RAD51B	SMC2	USP28
CASP3	EGFR	IGF1	OTUB1	RAD51C	SMC3	USP3
CCNA1	EME1	IGHMBP2	PALB2	RAD51D	SMC4	USP47
CCNA2	EME2	IKBKG	PAPD7	RAD52	SMC5	USP7
CCNB1	ENDOV	INIP	PARG	RAD54B	SMC6	UVRAG
CCND1	EP300	INO80	PARP1	RAD54L	SMG1	UVSSA
CCNE1	EPC2	INO80D	PARP2	RAD9A	SMUG1	VCP
CCNH	ERBB2	INO80E	PARP3	RAD9B	SMURF2	WDR16
CCNO	ERCC1	INTS3	PARP4	RASSF1	SOD1	WDR33
CDC14B	ERCC2	IRS1	PARP9	RB1	SP1	WDR48
CDC25A	ERCC3	JMY	PARPBP	RBBP4	SPATA22	WEE1
CDC25B	ERCC4	JUN	PCNA	RBBP7	SPIDR	WHSC1
CDC25C	ERCC5	KAT5	PLK1	RBBP8	SPO11	WRN
CDC45	ERCC6	KDM2A	PLK3	RBM14	SPP1	WRNIP1
CDC6	ERCC6L2	KIAA0101	PMS1	RBX1	SPRTN	WWP1
CDH13	ERCC8	KIAA0430	PMS2	RDM1	SSRP1	WWP2
CDK1	ESCO1	KIAA2022	PNKP	REC8	STAT1	XAB2
CDK2	ESCO2	KIF22	POLA1	RECQL	STRA13	XPA
CDK4	ESR1	KIN	POLB	RECQL4	SUMO1	XPC
CDK7	ETS1	KPNA2	POLD1	RECQL5	SUPT16H	XRCC1
CDKN1A	EXO1	LIG1	POLD2	RELA	SWI5	XRCC2
CDKN1B	EXO5	LIG3	POLD3	REV1	SWSAP1	XRCC3
CDKN2A	EYA1	LIG4	POLD4	REV3L	SYCP1	XRCC4
CDKN2D	EYA2	MAD2L2	POLDIP3	RFC1	TAOK1	XRCC5
CEBPG	EYA3	MBD4	POLE	RFC2	TAOK2	XRCC6
CEP164	EYA4	MC1R	POLE2	RFC3	TAOK3	XRCC6BP1
CEP170	FAM175A	MCM9	POLE3	RFC4	TCEA1	YY1
CETN2	FAN1	MCPH1	POLE4	RFC5	TDG	ZBTB32
CHAF1A	FANCA	MDC1	POLG	RFWD2	TDP1	ZFYVE26
CHAF1B	FANCB	MDM2	POLG2	RFWD3	TDP2	ZNF350
